# The WISTAH hand study: A prospective cohort study of distal upper extremity musculoskeletal disorders

**DOI:** 10.1186/1471-2474-13-90

**Published:** 2012-06-06

**Authors:** Arun Garg, Kurt T Hegmann, Jacqueline J Wertsch, Jay Kapellusch, Matthew S Thiese, Donald Bloswick, Andrew Merryweather, Richard Sesek, Gwen Deckow-Schaefer, James Foster, Eric Wood, Richard Kendall, Xiaoming Sheng, Richard Holubkov

**Affiliations:** 1 Center for Ergonomics, University of Wisconsin-Milwaukee, P.O. Box 784, Milwaukee, WI, 53201, USA; 2 Rocky Mountain Center for Occupational & Environment Health, Department of Family and Preventive Medicine, University of Utah, 391 Chipeta Way, Suite C, Salt Lake City, UT, 84108, USA; 3 Department of Physical Medicine and Rehabilitation, Medical College of Wisconsin, 8701 Watertown Plank Road, Milwaukee, WI, 53226, USA

**Keywords:** Epidemiology, Ergonomics, Cohort, Carpal tunnel syndrome, Strain index, TLV for HAL

## Abstract

**Background:**

Few prospective cohort studies of distal upper extremity musculoskeletal disorders have been performed. Past studies have provided somewhat conflicting evidence for occupational risk factors and have largely reported data without adjustments for many personal and psychosocial factors.

**Methods/design:**

A multi-center prospective cohort study was incepted to quantify risk factors for distal upper extremity musculoskeletal disorders and potentially develop improved methods for analyzing jobs. Disorders to analyze included carpal tunnel syndrome, lateral epicondylalgia, medial epicondylalgia, trigger digit, deQuervain’s stenosing tenosynovitis and other tendinoses. Workers have thus far been enrolled from 17 different employment settings in 3 diverse US states and performed widely varying work. At baseline, workers undergo laptop administered questionnaires, structured interviews, two standardized physical examinations and nerve conduction studies to ascertain demographic, medical history, psychosocial factors and current musculoskeletal disorders. All workers’ jobs are individually measured for physical factors and are videotaped. Workers are followed monthly for the development of musculoskeletal disorders. Repeat nerve conduction studies are performed for those with symptoms of tingling and numbness in the prior six months. Changes in jobs necessitate re-measure and re-videotaping of job physical factors. Case definitions have been established. Point prevalence of carpal tunnel syndrome is a combination of paraesthesias in at least two median nerve-served digits plus an abnormal nerve conduction study at baseline. The lifetime cumulative incidence of carpal tunnel syndrome will also include those with a past history of carpal tunnel syndrome. Incident cases will exclude those with either a past history or prevalent cases at baseline. Statistical methods planned include survival analyses and logistic regression.

**Discussion:**

A prospective cohort study of distal upper extremity musculoskeletal disorders is underway and has successfully enrolled over 1,000 workers to date.

## Background

Distal upper extremity musculoskeletal disorders (DUE MSDs) are common and result in large costs. They reportedly comprise 4% of all state workers’ compensation claims.[1-3] Of the more common DUE MSDs, carpal tunnel syndrome (CTS) is the most costly with an estimated average of $20,405 per claim [4] and aggregate costs of approximately US$2B annually [5]. Elbow MSDs are also common and the State of Washington has reported that elbow disorders accounted for the third highest incidence rate with 29.7 injuries per 10,000 full-time employees.[6] Despite high cost and prevalence, relatively poor epidemiological data and few prospective cohort studies have been reported.

CTS is the most studied of the DUE MSDs [4,7-18], and its relationship with work has been reported in many, mostly cross sectional studies and only a few longitudinal studies [7,8,16,18-27]. However, most occupational epidemiological studies of CTS reported have not used objective measures that included electrodiagnostic testing in case definitions. Rather they relied solely on symptoms or combinations of symptoms and physical examination findings (e.g., Hoffman-Tinel’s sign). [17] A majority of these studies also did not measure job physical factors [17]. Many did not control for common potential confounders noted above and/or did not include frequent follow-ups of the populations studied [7,17-20,22,24,25,28]. These weaknesses may limit the strength and impact of the available data on the etiology of CTS. In contrast with that disorder, epidemiological studies are far fewer and more limited in other distal upper extremity disorders including hand/wrist tendinoses [8,17,29-34], lateral epicondylalgia [8,17], medial epicondylalgia [7,8,17] and trigger digit [7,10,17,35-41]

There are several ergonomic job evaluation methods in use for DUE MSDs. These methods include the Strain Index (SI)[42], American College of Governmental Industrial Hygienists Threshold Limit Value for Hand Activity Level (TLV for HAL) [43], Rapid Upper Limb Assessment (RULA), and checklists of generic ergonomic factors. Except one, most studies have reported weak relationships for the TLV for HAL or no statistically significant relationships [14,16,22,25]. Others have reported significant relationships for the Strain Index. Individual job exposure factors such as force and repetition have also been associated with increased risk of carpal tunnel syndrome [9,11,14,19,20,23,44-52]. Thus, none of these methods have been viewed as fully validated, particularly lacking quantitative prospective cohort evidence [17]

This prospective cohort study’s alternate hypothesis is that there is a relationship between quantified ergonomic factors and subsequent risk of DUE MSDs after controlling for other risk factors and potential confounders Disorders targeted particularly include CTS and lateral epicondylalgia, although many other disorders and non-specific symptoms are included.

## Methods/design

This study is approved by the Institutional Review Boards of the University of Wisconsin – Milwaukee and the University of Utah (#03.02.059 and 11889 respectively).

**Figure 1:**
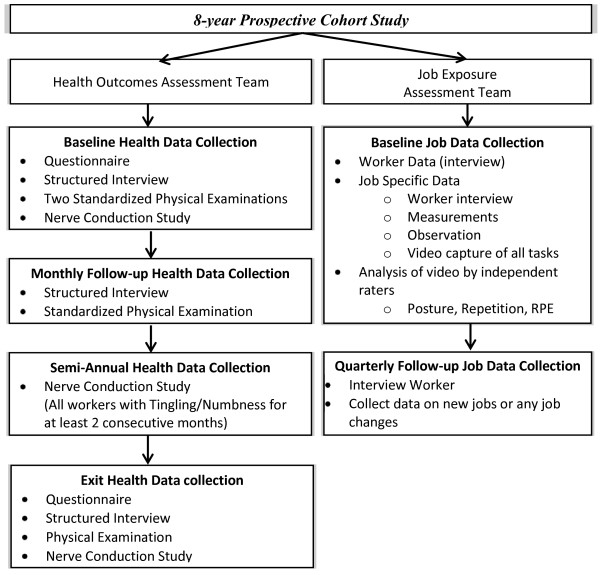
Data Collection Sequencing.

The design is a prospective cohort study. See Figure 1 for sequencing of data collection activities.

Workers for the study are recruited from 15 employers with 17 diverse production facilities located in Wisconsin, Utah and Illinois, USA. These employers include: (i) poultry processing, (ii) manufacturing and assembly of animal laboratory testing equipment, (iii) small engine manufacturing and assembly, (iv) small electric motor manufacturing and assembly, (v) commercial lighting assembly and warehousing, (vi) electrical generator manufacturing and assembly, (vii) metal automotive engine parts manufacturing (three facilities), (viii) plastic and rubber automotive engine parts manufacturing and assembly (ix) red meat processing, (x) apparel manufacturing, (xi) office work, (xii) cabinet manufacturing, (xiii) airbag manufacturing, (xiv) light valve assembly, and (v) small metal parts fabrication. Participating workers are paid regular wages by their employers. No incentives are paid for participation. Participants sign informed consent documents.

The goal is to enroll one-third of workers into each of three (low, medium and high) job physical demands groups. Eligible workers are required to be: (i) at least 18 years of age (due to job instability with youth), (ii) able to give informed consent, (iii) have no plans to retire or leave their employer within four years, (iv) able to speak either English or Spanish, and (v) free of major limb deformities and/or substantial amputations. Supervisors, maintenance/mechanics, and forklift truck drivers are not eligible due to probable frequent and unpredictable changes in job physical exposures as well as difficulty in efficiently videotaping these workers. With the exception of one setting (study sub-group), office workers are excluded as the ergonomic job evaluation methods are not primarily developed to measure these workers. However, workers transferring from eligible positions to office work are subsequently followed for development of MSDs.

The Health Outcomes Assessment Team is responsible for collecting health outcomes data, demographics, hobbies, physical activities outside of work, psychosocial and medical history data (see Additional file 1). The Job Exposure Assessment Team is responsible for collecting job physical exposure data (Additional file 2). These two different teams of researchers are blinded to each other.

## Baseline health outcomes data collection

After consent is obtained, the Health Outcomes Assessment Team administers questionnaires (see Additional file 1 for paper version) and structured interviews (see Additional file 1 for paper version) at baseline. The questionnaire and structured interview are computerized with skip sequences to speed administration and eliminate inappropriate questions (e.g., pregnancy for males or detailed questions on medical treatment when there had been no musculoskeletal disorder). Computerization is expected to improve data quality by ensuring standardized responses to questions and eliminating out-of-range responses. Both electronic and paper versions of the questionnaire are available in English and Spanish. Translators are used for questionnaires, structured interviews and examinations as required.

The questionnaire particularly includes medical health items and psychosocial factors (n = 266 items, see Additional file 1). Specific content areas include: (a) demographics (e.g., age, gender, and history of maximum body weight), (b) frequencies and durations of hobbies and outside of work activities, (c) medical history including diabetes mellitus, thyroid disorders, high blood pressure, high cholesterol, musculoskeletal disorders, inflammatory arthritis (including rheumatoid arthritis), and other relevant diseases, (d) psychosocial questions (e.g., depression, job satisfaction, family problems, supervisory and coworker support, etc.), and (e) other questions (e.g., sleeping patterns, smoking, alcohol consumption, family history of CTS). A total of 58 items that are generally classified as psychosocial, 16 of which are work organizational factors and some of these factors are assessed by the Job Exposure Assessment Team (see below). There are 24 items on job-related psychosocial factors (e.g., co-worker support) and 18 items on personal psychosocial factors (e.g., depression, anxiety). Other factors could not be included due to practical requirements to balance inclusion of factors against the significant increased time commitments to complete such instruments. Excessive time for completion is a limiting factor for companies’ participation.

A structured interview is administered by either Hand Therapists or Occupational Medicine Residents and includes a survey of symptoms required for diagnostic purposes (n = 483 items) (see Additional file 1 for paper version). The structured interview also utilized a body diagram to help localize symptoms. Symptoms included (a) current tingling and numbness in each digit (b) tingling and numbness in each digit in the past month, (c) current pain, ache, burning and/or stiffness in each body part (current MSDs are based on current symptoms plus physical exams), (d) pain, ache, burning and/or stiffness in each body part in past one month, (e) duration of pain, tingling and numbness in the past one month, and (f) prior history of specific disorders including CTS, lateral epicondylalgia (“epicondylitis”), medial epicondylalgia, de Quervain’s stenosing tenosynovitis, trigger digit/thumb, flexor wrist tendinosis and extensor wrist tendinosis. Symptoms and history of disorders were recorded for each hand separately.

A hand symptoms diagram is then completed by the worker for symptoms at the time of enrollment (see Additional file 1). Tingling and numbness are combined as one symptom, and pain of any type or quality (e.g., burning, stabbing) was the other symptom included. To facilitate both of the two subsequent physical examinations and diagnostic impressions, the computer program displays a summary of the body parts that have been symptomatic in the prior month.

Workers then undergo the first of two standardized physical examinations, one of the examinations by the same individual who administered the structured interview (see Additional file 1). Examinations include (a) observation of the DUE, (b) inspection of the DUE, (c) palpation, (d) range of motion, and (e) specific examination maneuvers. Signs of certain disorders such as rheumatoid arthritis, Dupuytren’s contracture, and Heberden’s and Bouchard’s nodes are recorded. Health team members are trained on the standard examinations through review of the videotape and practice in small group sessions until proficiency and consistency in accordance with the protocol are demonstrated. All maneuvers are performed in the first examination regardless of whether symptoms are present or not. These findings are recorded on paper to integrate information for the second physical examiner’s review (see Additional file 1).

The second physical examination is performed by board-certified Occupational Medicine physicians. There are two main information sources used to begin that examination: the symptoms summary page from the structured interview (above) and the results of the first examiner’s physical examination tests. The purpose of this examination is to confirm positive findings and to evaluate pertinent negatives. Additionally, this examination results in a diagnostic impression irrespective of the specific physical examination results and case definitions (see below).

Height and weight are measured in stocking feet in metric units (see Physical Examination form in Additional file 1). These are used to calculate body mass indices (BMI). If the weight exceeds 200 kg, two scales are used simultaneously with the sum of the two scales recorded. Blood pressure and heart rate are obtained in a seated position after a minimum of 5 min of rest, and most often after 20 min of rest (after completing the questionnaire). Automated cuff measures are utilized (Omron HEM-780). Wrist width and wrist depth are measured with calipers (Brown & Sharpe T20257 Digital Caliper) and recorded to the nearest tenth of a millimeter.

## Nerve conduction studies

All workers undergo a nerve conduction study of both hands, regardless of symptoms (see Additional file 1). These studies follow the recommendations of the American Association of Electrodiagnostic Medicine (2002) and are conducted by one of two board certified physiatrists who are blinded to the worker’s symptoms and job physical exposures. A TECA Synergy EMG machine is used. Hand dorsa temperatures are measured and a minimum hand temperature of 30 °C is assured with warming blankets. Latencies, amplitudes and stimulus parameters are recorded. Nerve conduction studies include median and ulnar nerve sensory and motor latencies as well as median and ulnar paired transcarpal short segments[53-57]. Transcarpal responses are recorded at the wrist with a 3 cm bar electrode with stimulation in the palm and an 8 cm distance between the cathode and E1 electrode. Median digital sensory studies are recorded in the long finger at 12 cm with ring electrodes with a 4 cm interelectrode distance. Ulnar digital sensory studies are similarly measured in the little finger at 12 cm with ring electrodes. Median motor studies are conducted at 6 cm with the E1 electrode over the thenar midpoint of the abductor pollicis brevis muscle and the E2 on the dorsal distal thumb phalanx. Ulnar motor conduction results are recorded at 6 cm with the E1 electrode over the midpoint of the abductor digiti minimi and the E2 on the dorsal distal fifth digit phalanx. Sensory peak latencies and motor onset latencies are recorded. Nerve conduction studies are tentatively classified as normal or abnormal (abnormal further categorized, as mild or moderate/severe) based on the criteria in Table 1. However, some workers undergo additional review by one physiatrist (JJW). These workers largely fall into two groups, (i) those missing a transcarpal ulnar latency and (ii) those whose pattern of abnormality/severity for transcarpal delta, sensory latency, and motor latency show an atypical pattern such as diffuse delayed conduction. Thus, final classifications of studies are: (i) normal, (ii) abnormal and consistent with median mononeuropathy (mild or moderate/severe), and (iii) abnormal and consistent with systemic neuropathy. Those workers showing signs of a systemic neuropathy (e.g., diabetic polyneuropathy) will be excluded from the subsequent analyses. Results of the nerve conduction studies are not communicated to the workers or management as studies do not support such communications [22] .

**Table 1:** Parameters for Nerve Conduction Study Classification

	**Classification**	Transcarpal Delta*	**Sensory Latency**	**Motor Latency**
	*Normal*	≤ 0.85 ms	≤ 3.70 ms	≤ 4.50 ms
*Abnormal*	Mild	> 0.85 ms	≤ 3.70 ms	≤ 4.50 ms
	Moderate	> 0.85 ms	> 3.70 ms	≤ 4.50 ms
	Severe	> 0.85 ms *or absent*	> 3.70 ms	> 4.50 ms

* Transcarpal Delta = (median nerve sensory latency – ulnar nerve sensory latency).

## Follow-up health outcomes

Monthly follow-ups are accomplished on-site by a health outcomes team member(s) who is assigned to that plant as the primary contact (see Additional file 1 for paper version). Besides administering monthly structured interviews, a primary function of these individuals is to maintain enthusiasm for the project. New symptoms and changes in symptoms are recorded with computerized structured interviews that include tingling and/or numbness which are recorded for each digit, as well as pain (see Additional file 1). Healthcare treatments obtained are also recorded. When new symptoms are reported, focused standardized physical examinations are performed to examine that body part using the same methods as the baseline examinations. Every six months, those workers with tingling/numbness for two or more consecutive monthly follow-ups are administered follow-up NCSs. Annually, important potential confounders (e.g., diabetes, self-reported weight) are queried and recorded. At least quarterly, a board-certified occupational medicine physician reviews the cases and provides a diagnostic impression.

The entire cohort under observation is periodically assessed with an abbreviated questionnaire, full length structured interview, physical examination and nerve conduction study. This has occurred at approximately 42 months into the study, and again at 78-84 months into the study.

The team attempts to ascertain why individuals drop out of the study. Most are due to layoffs or quitting current job to take a new job at a different company. Attempts are made to examine these workers and obtain a final diagnosis(es) for those who terminate prematurely. The last question for the monthly follow-up queries whether there have been any job changes, and positive responses are reported to the Job Exposure Assessment Team for repeat job measurements.

This is an observational study and therefore, the team does not treat the musculoskeletal disorders identified. Workers are encouraged to follow their employer’s procedures relating to reporting and securing medical care if the worker expresses a belief the musculoskeletal disorder requires healthcare. Workers are also referred to their provider if medical conditions such as hypertension are identified.

### Job physical exposure

#### Baseline job physical exposure data

Whenever new employees are recruited into the study, they undergo baseline job physical exposure data collection. Baseline data collection is performed within two months of the worker having their baseline health data assessment. Baseline data collection is broken into two major components: (i) job specific data collection and (ii) task specific data collection (Additional file 3). In this study *job* refers to the worker’s overall activities in a day. *Task* refers to specific, but unique, activities performed by the worker for a certain number of hours in a given day. A job can be comprised of a single task or multiple tasks (e.g. job rotation). Job rotation is fairly common in this study. The definition used of a sub-task is a unique combination of hand/wrist force, hand/wrist posture and number of exertions/cycle.

All job physical exposure data are collected at the facilities of the participating companies. Both quantitative and subjective measurements are recorded. All tasks performed by participating workers are recorded on digital videotape using hand-held video cameras. Tasks with cycle time ≤ 2 m are recorded for at least ten cycles and tasks with cycle time > 2 m are recorded for 20 to 45 m, ensuring at least one complete cycle recorded. Videos are taken using a single camera but from three different camera angles. Tasks with cycle time ≤ 2 m are videotaped for at least three cycles from each of three angles, and tasks with cycle time > 2 m are videotaped for at least 5 m from each of the three angles.

Data collection begins with the analyst introducing himself/herself to the worker. The analyst then observes the task for several cycles, videotapes the task, interviews the worker to collect relevant information about worker and tasks, provides analyst Borg CR-10 force ratings for DUE, obtains worker Borg CR-10 force ratings for DUE, and takes task physical exposure measurements (e.g., weights, pushing/pulling forces, grip and pinch strengths and matching forces, etc.). To ensure the video captured is an accurate representation of frequencies of different exertions, all videos are recorded in “real time,” without the worker being interrupted by the analysts.

### Job specific job physical exposure data-field measurements

Job specific data are collected to determine all different activities (tasks) performed by the worker and related information (Table 2) (Additional file 2). Job data include: (i) department and worker title, (ii) shift starting and ending time, (iii) different tasks performed by the worker, (iv) hours worked on each task/day, (v) task pace (self, line or piece rate), (iv) days worked per week, (v) prior work experience (# of years and Borg CR-10 rating for DUE), (vi) having a second job (# of years, hours/week, and dominant hand Borg CR-10 rating), (vii) Borg CR-10 ratings for applying a standardized 10-kg grip force, (viii) the worker’s maximum grip, lateral pinch and 3-point pinch strengths, and (ix) overall Borg ratings for distal upper extremity at the beginning and end of shift (Table 2).

**Table 2:** Physical Exposure at the Worker (Job) Level (measurements/observations in the field) (From Garg et al. 2010)

**Exposure Type**	**Measurements**
General	Department and worker title, shift length
Pace	Self, line, piece work
Job rotation	No. of tasks, duration of each task, title of each task
Prior work experience	Title, years on each job, and worker’s Borg CR-10 rating for DUE and each job
Second job outside facility	Title, years on second job, and worker’s Borg CR-10 rating for dominant hand and second job
Strength	Grip, lateral pinch and 3-point pinch for dominant hand
Fatigue	Overall worker DUE Borg CR-10 rating for the dominant hand at the end of the shift and beginning of the shift

Shift starting time, ending time, and days per week are recorded by interviewing the worker on the production floor. Next, workers are asked to briefly describe each of the tasks they perform as a part of their job held with the company. For those workers who work multiple tasks, each task and the total consecutive hours worked on the task are recorded. Workers are then asked to list their previous jobs held, the length of time in years that the job was held, and to provide a corresponding a Borg CR-10 rating for the DUE for each of the jobs listed. The first job listed is the “Current” job the employee held. Previous jobs are listed until the total previous employment duration sums to 10 years, or 5 previous (6 total, including the current) jobs are recorded, whichever occurs first. Secondary employment, or second jobs held outside the facility are recorded next. If the worker holds a second job, a brief description of the type of work performed is recorded. The worker is then asked how long they have held the second job, how many hours per week they work at the second job, and to provide an overall Borg CR-10 rating for the dominant hand corresponding to the second job.

Workers’ dominant handgrip, lateral pinch and 3-point pinch strengths are measured using grip and pinch dynamometers (3 trials for each measurement) (Additional file 2). These strengths are measured with the wrist in functional neutral position, upper arm hanging to the side and the lower arm horizontal and in functional neutral position (no forearm rotation). For grip strength measurements, the Jamar dynamometer setting two is used. Lastly, with regard to their primary job, the worker is asked to provide Borg CR-10 ratings for the level of physical stresses they feel on their distal upper extremity at the beginning of their work shift (about 30 min after they start their typical work day) and at the end of their work shift (about 30 min before the end of their typical work day). This information is gathered to estimate the accumulation of fatigue as a result of performing their various physical activities.

### Task specific job physical exposure data-field measurements

Data are collected for each task performed by a worker using Task Specific Data forms (Additional file 2). General observations include: (i) use of gloves (type of gloves and fit), (ii) room temperature, (iii) hand contact with a hot or cold object, and (iv) localized mechanical compression (body part and intensity). Specific information includes: (i) measured cycle time, (ii) analyst’s estimates of applied hand force for each hand and for each major task performed (Borg CR-10 ratings) (iii) weight of the workpiece or hand tool and center-of-mass offset of handtool (iv) matching grip, pinch and thrust forces for left and right hand (peak and typical values), (v) analyst ratings of applied hand/wrist forces for each hand (typical and peak values, Borg CR-10 scale), and (vi) worker ratings of applied hand/wrist forces for each hand (typical and peak values, Borg CR-10 scale).

Analysts provide their ratings first to avoid biasing their ratings based on the worker’s ratings. Similarly, workers are not allowed to see the analyst’s ratings. For the peak force rating, the Borg CR-10 rating is a representation of the force required to perform the most difficult sub-task of the task. Workers are asked to identify the most stressful sub-task they perform with regard to the distal upper extremity. Once identified, both the analyst and the worker provide their peak distal upper extremity force ratings for that task on the Borg CR-10 scale. The analyst and worker are also asked to provide “typical” force ratings for the task for each hand (Borg CR-10 scale). In those situations where applied hand forces vary during a cycle, both the analyst and the worker are asked to ignore the peak force exertions when assigning typical force rating. If the analyst or the worker feels that there is no appreciable variation in applied hand force then the typical and peak force ratings are the same.

Cycle time is determined using a stopwatch. Object weights are measured using a digital platform scale and pushing and pulling forces using a force gauge (Chattilon model # CSD250).

Scales on grip and pinch dynamometers are covered prior to measuring matching grip and pinch forces. Workers are asked to hold the grip/pinch dynamometer exactly in the same posture as that required when using the hand-tool. Then, the worker is asked to apply a force on the dynamometer equal to the force required to grip/pinch the hand-tool. Matching thrust forces are measured using a force plate. In case of a rotary hand tool (such as a nut runner or a screw driver) a thrust bearing is used between the tool and the force plate.

### Follow-up job physical exposure data collection

Every three months, a member of the job physical exposure team visits each employee. The job team has a computerized position form showing the analyst what jobs the worker is performing as of the last visit (3 months prior). The analyst carefully inspects all the jobs listed and determines if there are any material changes to the jobs. In most cases, the changes are minor and do not affect exposure levels. In cases where the job parameters substantially change, or the worker moves to a different job all together, the new/revised jobs are measured using all the job specific data forms (Additional file 2).

## Extraction of data from video analysis

Videos are analyzed frame by frame to determine intensity of force, temporal exertion requirements, hand/wrist posture, speed of work and type of grasp, etc. for each hand separately. Some of the analyses are at the task level while the others are at the sub-task level. Each task is divided into sub-tasks. As noted above, a sub-task is a unique combination of hand/wrist force, hand/wrist posture and number of exertions/cycle. During the analysis of videotape if there is a change in either hand/wrist force, hand/wrist posture or number of exertions/cycle a new sub-task is created. Thus a task consists of multiple sub-tasks, each sub-task defined by intensity of exertion (force), number of exertions/cycle, duration per exertion, hand/wrist posture and speed of exertion. (Additional file 2). All these variables are determined by analysts. Table 3 summarizes the job physical exposure task level.

**Table 3:** Physical Exposure at the task level (measurements/observations in the field (m) and from videotape analysis (v))

**Variable**	**Measurement**
Cycle Time (seconds)	SI definition (v)
Force	Analyst DUE force rating (Borg CR-10) (1) Peak force (m), (2) Typical force (m), (3) Overall force (m), analyst judgment (Moore and Garg, 1995) (4) Overall force using an algorithm (Garg and Kapellusch)
	Worker DUE force rating (Borg CR-10) (5) Peak force (m), (6) Typical force (m)
	Matching force) (7) Grip force (m), (8) Pinch force (m), (9) Thrust force
	Measurement of weights and forces (10) Object/tool weight and Center mass offset (m), (11) Pushing/pulling force (m)
Repetition	(1) HAL Rating (v) (Latko 1997)
	(2) No. of exertions/min (SI) (v) (Moore and Garg 1995)
Duration of Exertion	(1) % duration of exertion (v) (Moore and Garg, 1995)
	(2) Total duration of exertion (seconds/min) (v)
Exposure/day (hours)	Supervisor/worker (m)
Hand/wrist Posture	Posture categories (v)
	(1) Wrist flexion: <30, 30-50, >50
	(2) Wrist extension: <30, 30-50, >50
	(3) Ulnar deviation: <10, 10-25, >25
	(4) Radial deviation: <5, 5-25
	(5) No. of exertions in each category
	(6) % of cycle time in each category
	(7) Peak force posture categories
	(8) Overall SI posture (Moore and Garg, 1995)
Elbow Posture	(1) Extension (v) (a) < 70 and (b) > 135
	(2) No. of exertions (v)
	(3) % cycle time (v)
	(4) Forearm position (v): (Neutral, prone, supine)
Speed of work	Using the Strain Index method (Moore and Garg, 1995).
Forearm Rotation	% of cycle time with forearm rotation (v) > 45
Grip/pinch	(1) Type of grasp (v): (a) power, (b) oblique, (c) palmer grip, (d) hook grip
	(2) Type of pinch (v): (a) palmer pinch, (b) -point, 2-point, (c) lateral, (d) 2-finger scissor;
	(3) Grip/pinch span (v)
	(4) % cycle time in each type of grasp/pinch (v)
Localized Mechanical Compression	(1) Body part (v)
	(2) Category (v): (a) Negligible, (b) moderate, (c) severe)
	(3)No. of exertions/min.(v)
	(4)% of cycle time(s) (v)
Hand as hammer	(1) Category (v): (a) Negligible, (b) moderate, (c) severe)
	(2) No. of exertions/min.(v)
Tool kicks	(1) Category (v): (a) Negligible, (b) moderate, (c) severe)
	(2) No. of exertions/min.(v)
Gloves	(1) Type (m)
	(2) Fit (m)
Exposure to hand/arm vibration	% of cycle time spent in (a) negligible, (b) visible and (c) severe hand/arm vibration

In summary, all field measurements are either at the worker level or task level. All videotape analyses are either at the task level or sub-task level. These measurements are combined to quantify job physical exposures at the worker level, task level and sub-task level.

## Job physical exposure data analyses

All measured variables in the field and those obtained from videotape analyses are entered into a central database. The job physical exposures are calculated at sub-task, task and job (worker) levels. For each task average and peak force, overall force, total number of exertions/min, average and worst hand wrist posture, and several other variables listed in Table 3 are determined. Exposures at the task level are used to assign exposure at the worker level as discussed later under assigning exposure at the worker level.

## Classifications of TLV for HAL and the strain index

**Figure 2:**
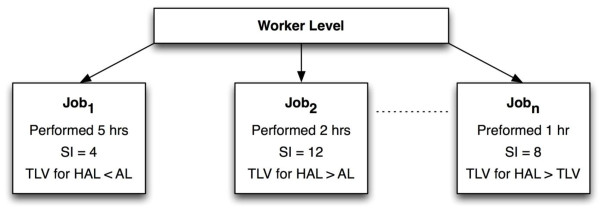
**Example of a Worker’s Job for Illustrating Exposure Classification.** Task 1 represents the longest task performed in the day and thus it is the typical exposure for the Strain Index (SI) and Threshold Limit Value for Hand Activity Level (TLV for HAL). Task 2 represents the peak exposure for the SI and Task n represents the peak exposure for the TLV for HAL as it has the highest threshold limit value, which exceeds the TLV.

A combination of the peak force rating (Borg CR-10) and HAL rating is used to determine TLV for HAL classifications (below the Action Limit (AL), between the AL and TLV, and above the TLV) using the ACGIH (2002) method. These are referred to as TLV for HAL categories 1, 2 and 3 respectively. Two different peak force ratings are used (worker peak force rating and analyst peak force rating) to determine TLV for HAL classification. The analyst’s overall force rating (intensity of exertion converted from Borg CR-10) is used to calculate the Strain Index score (SI score). The analyst’s overall force ratings on Borg CR-10 scale are converted into intensity of exertion ratings for the SI calculations by matching verbal anchors from the Borg CR-10 and SI intensity of exertion scales. Specifically, analyst’s overall CR-10 ratings from 0-2 (light), are assigned an SI intensity of exertion rating of 1 (light). For CR-10 ratings of 3 (moderate)-4 (somewhat hard) an SI rating of 2 (somewhat hard) is assigned. TLV for HAL and SI scores are calculated for each worker and task performed throughout the follow-up period. Peak force, highest repetition and worst posture are determined from the tasks that result in the highest exposure for these variables (peak exposure, Figure 2). The cut points used for the TLV for HAL are those prescribed by the ACGIH (2002). These were (peak force/(10-HAL)) < 0.56 for below Action Limit (AL) and (peak force/(10-HAL)) > 0.78 for above Threshold Limit Value (TLV). The cut point used for the Strain Index was Strain Index score (SI score) ≤ 6.1 and SI score > 6.1 (Moore et al. 2006).

## Assigning exposure at the worker level

Twenty percent of the workers perform more than one task during their workday or rotate jobs. As there is no consensus method to quantify job exposures for a worker who performs two or more tasks (Garg and Kapellusch 2009a), the TLV for HAL and SI score are summarized in two different ways. These are “typical exposure” and “peak exposure.” Typical exposure is defined as the exposure from a task the worker performs most of the time. Peak exposure is defined as exposure from a task that produces the highest physical exposure measures (SI score or TLV for HAL). See Figure 2 for graphic representations. It should be noted that the peak SI score and peak value for TLV for HAL might be from different jobs.

## Sub-task analyses

Analyses are also planned at sub-task levels. Each task is divided into sub-tasks and each sub-task is described by its force, exertions per minute, hand/wrist posture, duration per exertion, speed of work and hours/day (Table 4). As a simple example, consider a worker who does assembly of transformers for five hours/day and operates a grinding machine for three hours/day. Assembly of a transformer requires 2 exertions (cutting wire) at 60% MVC (2 s each, 45° wrist extension), 3 exertions (wrapping wire) at 35% MVC (1.5 s each, 30° wrist flexion), and 5 exertions (driving screws using a powered screw driver) (3 s each and neutral wrist); a total of 10 exertions in a 30 s cycle. This job has three tasks: cutting wire, wrapping wire and driving screws. All three tasks require different force (%MVC), durations of force and hand wrist posture. The dilemma is how to combine physical exposures from these three tasks to represent exposure at the job level. Further how to combine exposure from assembly job and grinding job to quantify exposure at the worker level? We propose to apply innovative methods that we have developed to quantify job physical exposure from tasks with multi-subtasks and job rotation (Garg and Kapellusch 2009). We plan to develop integrated exposure metrics that can address multi-subtask tasks using a weighting system that scores the subtask with the highest physical exposures first and then adds incremental weights for other lower exposure subtasks.

**Table 4:** Physical exposure at the sub-task level from videotape analysis (Example Assembly of transformers)

**Task Description**	**Force (Borg CR-10)^1^**	**Number of exertions/min (SI)^2^**	**Hand/wrist Posture (SI)^2^**	**Duration/exertion (seconds)**	**Speed (SI)^2^**
Cutting wire	7	2	Bad	2	Fair
Wrapping Wire	4	3	Fair	1.5	Fair
Driving screws	2	5	Fair	3.0	Fair

^1^Borg CR-10 scale, ^2^Strain Index definition.

## Case definitions

Case definitions utilizing the structured interview, physical examination and/or nerve conduction study results were defined for all outcomes (see Table 5). No acute injury events whether at home or work (e.g., slip, trip, fall) will be included in the analyses for risks of diseases although they will be summarized. Included in the case definitions will be those non-acute injury events that the worker believes are either due to work or for which the cause is given by the worker as unknown. For primary outcomes, disease recurrence will be excluded, although for some outcomes and analyses, a symptom-free interval of at least 3 months will be required prior to eligibility to develop a recurrent case.

**Table 5:** Case Definitions for Musculoskeletal Disorders

**Disease criteria for case**	**Exclusions & Right Censor Conditions**
**Carpal Tunnel Syndrome:**	**Exclude from Consideration if:**
Case if meets: (1 + 2 + 3 + 4) OR 5	· had both tingling/numbness and an abnormal nerve study at baseline (met the prevalence case definition, hand specific^1^)
1. Numbness/Tingling (N/T) in 2 or more median nerve served digits (thumb, index, middle finger and/or ring finger) for ≥25% of days and/or nights on at least 2 consecutive monthly followups (from monthly follow-up interview). Note: N/T at baseline counts as one of two consecutive followups).	
	· has evidence of systemic neuropathy (determined by JJW, censor for all CTS analyses)
	· had prior Carpal Tunnel Release surgery (hand specific^1^)
	· had prior diagnosis of CTS by a Physician (hand specific^1^)
2. Abnormal nerve conduction study consistent with median mononeuropathy at the wrist (from baseline, or semiannual NCS) that was independently interpreted by a blinded, board certified physical medicine and rehabilitation physician (JW).	
	· had prior injection for CTS (hand specific)
	· has amputation of second or third digits at MCP or PIP in either hand (censor for all CTS analyses)
	**Right Censor if:**
3. Time difference between + (positive) NCS and consecutive N/T followups must occur within 6-months)	· becomes CTS incident case (hand specific1)
4. Automatically a case if has surgery for CTS, provided the surgery cause is said to be “work-related” or “unsure”) and review by physician (KTH) suggests CTS.	· leaves the study permanently (non-case)
**Lateral Epicondylalgia:**	**Exclude from Consideration if:*all exclusions are hand specific***^***1***^
Case if meets: (1 + 2 + 3) OR 4	
1) Lateral elbow pain on interview present for ≥ 25% of days since last follow-up (from monthly follow-up interview).	· met the case definition at baseline
	· had prior lateral elbow surgery
	· had prior elbow surgery of unknown type
2) “Pain” upon palpation of 1 or more of 6 lateral tender points (from monthly follow-up physical exam).	· had prior diagnosis of lateral epicondylalgia
	· had prior treatment for lateral epicondylalgia
3) Automatically a case if have surgery or injection for lateral epicondylalgia, provided the surgery cause is said to be “work-related” or “unsure”) and review by physician (KTH) suggests lateral epicondylalgia.	
	· had prior radial nerve pain
	**Right Censor if:**
	· becomes Lateral Epicondylalgia incident case (hand specific^1^)
	· suffers an elbow injury (i.e. accident, fall, etc..) (hand specific1, non-case)
	· permanently leaves the study (non-case)
**Medial Epicondylalgia:**	**Exclude from Consideration if:*all exclusions are hand specific***^***1***^
Case if meets: (1 + 2 + 3) OR 4	
1) Medial elbow pain on interview present for ≥ 25% of days since last follow-up (from monthly follow-up interview).	· met the case definition at baseline
	· had prior medial elbow surgery
	· had prior elbow surgery of unknown type
2) “Pain” upon palpation of 1 or more of 2 medial tender points (from monthly follow-up physical exam).	· had prior ulnar neuropathy or cubital tunnel surgery, OR clinical impression of ulnar neuropathy.
3) Automatically a case if have surgery or injection for medial epi, provided the surgery cause is said to be “work-related” or “unsure” and review by physician (KTH) suggests medial epicondylalgia.	· had prior diagnosis of medial epicondylalgia
	· had prior treatment of medial epicondylalgia
	**Right Censor if:**
	· becomes medial epicondylalgia incident case (hand specific^1^)
	· suffers an elbow injury (i.e. accident, fall, etc..) (hand specific1, non-case)
	· permanently leaves the study (non-case)
**deQuervain’s Tenosynovitis:**	**Exclude from Consideration if:*all exclusions are hand specific1***
Case if meets: (1 + 2 + 3 + 4) OR 5	
1. Radial wrist pain for ≥ 25% of days since last follow-up (from monthly follow-up interview).	· met the case definition at baseline
	· had prior deQuervain’s surgery
	· had prior deQuervain’s treatment (injection)
2. 1st extensor compartment tenderness (from monthly follow-up physical exam).	
	· had prior deQuervain’s diagnosis
3. Positive Finkelstein test (active) (from monthly follow-up physical exam).	· has CMC/Wrist/MCP arthritis at baseline (or prior)
4. Automatically a case if have surgery or injection for deQuervain’s, provided the cause is said to be “work-related” or “unsure” and review by physician (KTH) suggests deQuervain’s.	**Right Censor if:**
	· becomes deQuervain’s incident case (hand specific^1^)
	· suffers a wrist injury (i.e. accident, fall, etc..) (hand specific1, non-case)
	· develops CMC/Wrist/MCP arthritis (hand specific1, non-case)
	· permanently leaves the study (non-case)
**Extensor Tendinosis (compartments 2-6)**	**Exclude from Consideration if:*all exclusions are hand specific***^***1***^
Case if meets: (1 + 2 + 3 + 4) OR 5	
1. Dorsal wrist pain for ≥ 25% of days since last follow-up.	· met the case definition at baseline
	· had prior wrist extensor tendinosis surgery
2. 2-6 extensor compartment tenderness.	
	· had prior wrist extensor tendinosis treatment (injection)
3. Positive resisted wrist extension	
	· had wrist arthritis at baseline (or prior)
4. Automatically a case if have surgery or injection for extensor tendinosis, provided the cause is said to be “work-related” or “unsure” and review by physician (KTH) suggests extensor tendinosis.	**Right Censor if:**
	· becomes wrist extensor tendinosis incident case (hand specific^1^)
	· suffers a wrist injury (i.e. accident, fall, etc..) (hand specific^1^, non-case)
	· develops wrist arthritis (hand specific^1^, non-case)
	· permanently leaves the study (non-case)
**Digital Flexor Tendinosis**	**Exclude from Consideration if:*all***
Case if meets: (1 + 2 + 3 + 4) OR 5	***exclusions are hand specific***^***1***^
1. Volar wrist pain – from Hand Pain Diagram	· met the case definition at baseline
	· had prior flexor tendinosis surgery
2. Digital flexor tendon tenderness (from monthly follow-up physical exam).	
	· had prior flexor tendinosis treatment (injection)
3. No numbness/tingling in digits 1-4 (from monthly follow-up interview).	
	· had wrist arthritis at baseline (or prior)
	**Right Censor if:**
4. Automatically a case if have surgery or injection for digital flexor tendinosis, provided the cause is said to be “work-related” or “unsure” and review by physician (KTH) suggests digital flexor tendinosis	
	· becomes flexor tendinosis incident case (hand specific1)
	· suffers a wrist injury (i.e. accident, fall, etc..) (hand specific1, non-case)
	· develops wrist arthritis (hand specific1, non-case)
	· permanently leaves the study (non-case)
**Trigger Finger/Trigger Thumb**	**Exclude from Consideration if:*all exclusions are hand specific***^***1***^
Case if meets: (1 + 3) OR (2 + 3) OR 4	
1. Pain in the finger (from both monthly follow-up physical exam and interview) AND Focal tenderness over A-1 pulley	· met the case definition at baseline
	· had prior trigger finger/thumb
	· had prior finger/hand surgery
2. Demonstrated triggering (from monthly follow-up physical exam OR monthly interview).	· had prior treatment for trigger finger/thumb (injection)
	· had MCP/finger OA at baseline
3. Automatically a case if have surgery or injection for trigger finger, provided the cause is said to be “work-related” or “unsure” and review by physician (KTH) suggests trigger finger.	**Right Censor if:**
	· becomes trigger finger/thumb incident case (hand specific^1^)
	· suffers a hand/finger injury (i.e. accident, fall, etc..) (hand specific1, non-case)
	· permanently leaves the study (non-case)
**Non-Specific Pain**	**Exclude from Consideration if:*all exclusions are hand specific***^***1***^
Case if meets: (1 + 3) OR (2 + 3)	
1) Pain in DUE with intensity ≥ 6 for ≥ 25% of days since last follow-up that is NOT associated with a specific disorder.	· met the case definition for non-specific pain at baseline
	· met the case definition for any specific disorders at baseline
2) Pain in DUE of any intensity AND taking medication for pain.	
3)	a. Carpal Tunnel Syndrome
	b. Lateral Epicondylalgia
	c. Medial Epicondylalgia
	d. deQuervain’s
	e. Extensor Tendinosis
	f. Digital Flexor Tendinosis
	g. Trigger Finger/Trigger Thumb
	**Right Censor if:**
	· becomes a non-specific pain incident case (hand specific^1^)
	· becomes an incident case for ANY specific disorder
	a. Carpal Tunnel Syndrome
	b. Lateral Epicondylalgia
	c. Medial Epicondylalgia
	d. deQuervain’s
	e. Extensor Tendinosis
	f. Digital Flexor Tendinosis
	g. Trigger Finger/Trigger Thumb
	· suffers a DUE injury (i.e. accident, fall, etc..) (hand specific1, non-case)
	· permanently leaves the study (non-case)
**Aggregate Disorders**	**Exclusions from Consideration:**
Case if meets: 1	· Subjects are excluded from becoming a case under specific disorders based on the specific exclusion criteria above. Note: Subjects may still be eligible to become an aggregate disorder case despite being ineligible under certain disorders. (e.g. a subject that is excluded from becoming a case for trigger finger/trigger thumb, DeQuervain’s, and lateral and medial epicondylalgia, may still become a case for CTS, extensor, or digital flexor tendinosis, and is therefore still eligible to become a case for aggregate disorders.)
2. Meets any of the following case definitions as defined above:	
a. Carpal Tunnel Syndrome	
b. Lateral Epicondylalgia	
c. Medial Epicondylalgia	
d. deQuervain’s	
e. Extensor Tendinosis	
f. Digital Flexor Tendinosis	
g. Trigger Finger/Trigger Thumb	
Note: person level based on development of any of above disorders in *either* hand.	
	· If a subject is not eligible to become a case
	for any of the 7 specific disorders listed to the left, the subject is excluded from aggregate disorders
	**Right Censor if:**
	· becomes aggregate disorder incident case (hand specific^1^)
	· suffers a hand/wrist/elbow injury (i.e. accident, fall, etc..) (hand specific1, non-case)
	· permanently leaves the study (non-case)

^1^hand specific, out for person level analyses, out for dominant hand if dominant hand affected.

The case definition for carpal tunnel syndrome is tingling/numbness in at least two median nerve served digits (thumb, index, middle and ring fingers) present for at least 25% of the time for at least 2 consecutive monthly follow-up periods plus an abnormal nerve conduction study consistent with carpal tunnel syndrome. Those cases meeting the case definition at baseline, having been previously diagnosed as having CTS, or having undergone carpal tunnel surgical release are excluded from eligibility for becoming an incident case.

The primary case definition for lateral epicondylalgia is pain in the lateral elbow (from the body diagram) plus pain on palpation of at least one of the six tender points from the physical examination (see Additional file 1). A secondary case definition includes at least one positive physical examination maneuver of resisted wrist extension or resisted middle finger extension.

The physicians’ diagnostic impressions are not used in the primary analyses. However, they are planned to be used separately to address the relative value of the diagnostic impressions versus the case definitions

At each monthly health outcomes follow-up, the final question asked of the worker is whether the job has changed. Job changes identified by the worker are referred to the Job Exposure Assessment Team for evaluation and potential re-measurements (see above).

## Statistical analyses

Key statistical analyses in this study include: 1) Determination of prevalence for specific musculoskeletal disorders, 2) Calculation of incidence rates for disorders, 3) Evaluation of risk factors for disorders, 4) Evaluation of interactions between various risk factors and specific disorders (e.g., carpal tunnel syndrome and lateral epicondylitis), 5) Analyses of the performance of existing ergonomic models, and 6) Building models for predicting risk(s) of DUE MSDs. Data will be analyzed in SAS 9.2 (SAS Institute, Cary, North Carolina, USA). Significant associations will be reported based two-sided statistical significance with an alpha of 0.05.

The unit of analysis in the study for primary outcomes is individuals. The final point prevalence of specific distal upper extremity musculoskeletal disorders at baseline will be calculated. Baseline prevalence of specific musculoskeletal disorders including lateral epicondylalgia, medial epicondylalgia, deQuervain’s, trigger digit and hand/wrist tendinosis will be aggregated into baseline prevalence of non-CTS DUE MSDs. Similarly, past history of specific DUE MSDs will be aggregated into a lifetime prevalence estimate of these disorders. The baseline prevalence of a specific disorder will be calculated and those worker’s hands will subsequently be excluded from incidence analyses for that specific disorder.

Some health outcomes, such as prevalence and incident cases of lateral epicondylalgia, are binary variables and will be analyzed using logistic regression models for prevalence and proportional hazards regression models for incidence. Other variables, such as impairment or severity, may be ordinal categorical or continuous (e.g., number of lost or restricted workdays) and will be analyzed using corresponding nonparametric techniques. Risk factors will be grouped according to nature (individual, psychosocial, and job physical factors) and introduced into the models. Associations between predictor variables (including existing job analysis methods) and health outcomes will initially be evaluated using univariate methods. Variables with meaningful evidence of association to the health outcomes (generally existing at p < 0.20) will be considered for inclusion in multivariate models.

Incidence rates will be assessed using approaches parallel to those from the baseline analyses. Cumulative rates will be calculated using subjects remaining in the study at that time with known status. Information from subjects withdrawing from the study will be incorporated by calculating Kaplan-Meier rates of freedom from symptoms/disorder at a particular point in time using survival analysis methods.

Unadjusted univariate hazard ratios (HR) for incident cases of specific disorders and 95% confidence intervals will be determined for TLV for HAL, Strain Index score, individual ergonomic variables (e.g., force, repetition, posture) and relevant covariates using Cox proportional hazard regression with time varying covariates [58] in SAS version 9.2 using the PHREG statements [59].

Potential covariates (see Table 6) will be grouped and evaluated for association with incident cases of the musculoskeletal disorder in survival analyses prior to creating multivariate models. Multivariate analyses will be performed for job physical factors (e.g., force, repetition, hand/wrist posture), as well as the TLV for HAL and Strain Index score. Covariates will be selected (from worker demographics, hobbies and physical activities outside of work, psychosocial factors, baseline prevalence of distal upper extremity musculoskeletal disorders other than the disorder being analyzed and medical history) based upon p ≤ 0.20 and biological plausibility.

**Table 6:** Potential Covariates Considered for Multivariate Analyses of MSDs

**Demographic**	**DUE MSDs other than**
Age	**CTS**
Gender	Baseline prevalence
Handedness	Lifetime cumulative
Currently smoking	prevalence
Ever smoked	**Hobbies and Activities**
Alcohol	Aerobics
Marital status	Bicycling
Family history of CTS (blood relatives)	Running
	Swimming
Pregnancy	Walking
**Anthropometric**	Weightlifting
Body mass index	Baseball
**Past Medical History**	Basketball
Diabetes mellitus	Football (American)
Gout	Racquetball
High blood pressure	Snow skiing
High cholesterol	Tennis
Rheumatoid and other	Water skiing
Inflammatory arthritis	Car maintenance
Osteoarthrosis	Motorcycling
Kidney failure	Piano
Thyroid problem	Remodeling
Wrist fracture	Snow shoveling
**Psychosocial**	Snowmobiling
General health compared to others	Vibrating tools
Family problems	Woodworking
Feelings of depression	
Feel mentally exhausted	
Feel physically exhausted	
Employer cares	
Get along with coworkers	
Job satisfaction	
Recommend job to others	
Supervisor appreciation	
Would take their job again	

Separate proportional hazard regression models will be fitted for each of the job physical exposure tools (SI, TLV for HAL) as well as individual job physical exposure variables such as peak force and repetition. We will then assess the hazard ratio, with 95% confidence intervals, for each factor in a proportional hazard model adjusted for confounding variables.

Changes in exposure level occurring in the course of the study will be incorporated in some of these analyses. Additional analyses to model associations with events occurring more than once in the same individual over the study period (e.g., elbow pain that recurs 6 months later) using the Andersen-Gill independent increment method [60] and other approaches are planned. These other approaches involve fitting a basic proportional hazard model that ignores potential correlations to an appropriately define risk set, and then implementing a robust covariance estimate to adjust for correlation between events occurring in the same subject. [61] Transformation or categorization of a predictor is an option if there are problems with model fit. Risk factors that are significant, or show a strong trend, after adjustment, will also be considered as candidates for ergonomic models incorporating the “best” independent predictors of events.

For assessment of the predictive performance of existing ergonomic models (particularly Strain Index, ACGIH TLV for HAL, and the Rapid Upper Limb Assessment), the incidence data will serve as the gold standard against which the operant characteristics such as sensitivity and specificity will be analyzed [42,62].

For assessing the appropriateness of analysis approaches, whether dropouts are independent of study outcome will be assessed (“data missing completely at random, MCAR”, [63]). If the missing completely at random assumption is tenable, then analyses making use of available data will generally produce valid inferences. Survival analysis methods will facilitate the use of available follow-up data in subjects who drop out of the study, under the assumption of noninformative censoring.

These analyses involve examination of several indices of exposure and several measures of two main outcomes, leading to potential for chance associations due to multiple statistical tests [64-69]. This study will use a limited number of “primary analyses” that use uncorrected significance levels, given that the intended (and actual, if different) analysis plan for the study is clearly stated in reports and publications. Exploratory analyses, such as *post hoc* stepwise model building, will be reported as such.

The assessment of interactions will be performed by evaluating combinations of selected risk factors. Combinations of job physical factors, particularly between pairs of force, repetition and posture will be evaluated. Individual risk factors (particularly obesity and diabetes mellitus) as well as combinations of job physical demands with individual risk factors (i.e. force and obesity, etc.) will be evaluated.

## Discussion

A large, multi-center prospective cohort study is underway to quantify risks of distal upper extremity musculoskeletal disorders and to assess the performance of existing ergonomic job evaluation methods. This cohort study is addressing numerous weaknesses of prior research studies including use of: 1) prospective methods, 2) multi-center with two diverse states, 3) computerized data collection methods of questionnaires and structured interviews to assure data collection, 4) standardized physical examinations that include one comprehensive examination and one symptom focused examination, 5) nerve conduction studies in all subjects, 6) ability to exclude pre-existing or prevalent cases at baseline, 7) blinding of Health Outcomes Assessment and Job Exposure Assessment Teams, 8) additional blinding of the nerve conduction studies to symptoms, 9) monthly follow-ups of the cohort individualized quantification of job physical exposures, 10) heavy reliance on objective measures of exposure, 11) methods to account for job rotation and multiple task analyses, 12) careful case definitions, and 13) plans to evaluate interactions between and among job, individual and psychosocial factors.

Subjects are being enrolled from 15 employers in 17 different employment settings and over 1,000 subjects have been enrolled to date. The overall participation rate is not known as several plants invite workers to participate in a meeting from which enrollments ensue, thus the total target population in those plants is unknown. The highest participation rate in one plant with individualized enrollment processes is 96.0%. In plants enrolling in group meetings, approximately 75% of subjects attending those meetings enroll.

The cohort has been followed for several years. The success rate in contacting the cohort on a monthly basis has been calculated at 83.5%. The success rate of identifying reasons for absences is nearly 100%. Reasons for worker absences are tracked, and include: (i) vacation (most common), (ii) illness, (iii) leave of absence (e.g., funeral), and in a few cases (iv) absence due to surgery or treatment for a musculoskeletal disorder at any given observation period.

Study limitations include that workers are primarily from manufacturing environments, thus the results might not be applicable to other environments. Some of the commonly reported risk factors such as diabetes mellitus, thyroid disease and pregnancy are likely to be underpowered due to limited sample sizes for those conditions as this is a convenience sample that targeted one-third low, medium and high job physical demands. Also, the numbers of psychosocial questions was somewhat limited by the practical limits of time allowed by participating companies for enrollment of subjects and may be insufficient for some psychosocial variable domains.

## Competing interests

The authors have no competing interests.

## Authors’ contributions

AG serves as study Principal Investigator (PI), designed the study, is responsible for all phases of the project, serves as the lead for the Job Exposure Assessment Teams (JEATs) and helped draft the manuscript. KH helped design the study, serves as the PI for the University of Utah, leads the Health Outcomes Assessment Teams and drafted the manuscript. JJW serves as the PI for the Medical College of Wisconsin, is responsible for the nerve conduction studies and leading the team conducting those studies. AG, DB, JK, AM, RS coordinate the ergonomic measurements and the Job Exposure Assessment Team’s activities. KH, GD, JF, EW coordinate the health outcomes measurements and the Health Outcomes Assessment Team’s activities. JJW and RK perform the nerve conduction studies. MT, XS, RH coordinate the data and statistical management team. All authors read and approved the final manuscript.

## Pre-publication history

The pre-publication history for this paper can be accessed here:

http://www.biomedcentral.com/1471-2474/13/90/prepub

## Supplementary Material

Additional file 1 **Appendix B:** Health Outcomes Data Collection Instruments. Click here for file

Additional file 2 **Appendix C:** Job Physical Exposure Data Collection Instruments. Click here for file

Additional file 3 **Appendix A:** Job Classification (PDF 215 kb) Click here for file
