# Supplementary material for: The WISTAH hand study: A prospective cohort study of distal upper extremity musculoskeletal disorders
Source: BMC Musculoskelet Disord. 2012 Jun 6;13:90. doi: 10.1186/1471-2474-13-90 (PMC3476983; doi:10.1186/1471-2474-13-90)

Appendix B:

Health Outcomes Data Collection Instruments

B.1 Questionnaire ....
B.2 Structured Interview...
B.3 Sectioned Upper Extremity and Upper Torso Body Diagram ....
B.4 Hand and Digit Diagram..
B.5 Physical Examination Form...
B.6 Nerve Conduction Form ....
B.7 Pain Scale...

Appenidix B.1: Questionnaire

Upper Limb Musculoskeletal Disorders:
Baseline Questionnaire

Directions:

Please answer each question by pointing the arrow with the mouse and clicking with you index finger to either mark “yes” or “no” or to fill in a blank. If you need help or have any questions please ask one of our research assistants. We’re happy to help!

Code # ________________________
Date: _________________________

Upper Limb Musculoskeletal disorders: Baseline Questionnaire

1. Your Company's name: ______________________________________
2. Job Title / Department: _________________________________
3. Age: ____._ (in tenths of a year)
4. Gender: ____Male ____Female
5. Are you:
   ____ Right-handed
   ____ Left-handed
   ____ Use both hands equally
6. Are you planning on leaving or retiring (from this company) in the next 3 years? ______ Yes ______ No *If yes, please ask the research assistant before going on.*
7. How long have you worked in your current job? _______years ________months

8. Do you get any of the following types of exercise (outside of work) on a regular basis?

Type of Exercise	Yes	No	Number of months per year	Average number of times per week	Average number of minutes each time
Aerobics, Jazzercise			Months	Per week	Minutes
Running, Jogging			Months	Per week	Minutes
Walking			Months	Per week	Minutes
Bicycling			Months	Per week	Minutes
Swimming			Months	Per week	Minutes
Weight Lifting			Months	Per week	Minutes
Baseball			Months	Per week	Minutes
Basketball			Months	Per week	Minutes
Football			Months	Per week	Minutes
Racquetball			Months	Per week	Minutes
Handball			Months	Per week	Minutes
Tennis			Months	Per week	Minutes
Snow Skiing or Snowboarding			Months	Per week	Minutes
Water Skiing or Wave Runner			Months	Per week	Minutes
Other (please list)			Months	Per week	Minutes

9. Do you have hobbies that involve repetitive use of your hands (outside of work) such as any of the following?

Type of Hobby	Yes	No	Number of months per year	Average number of times per week	Average number of minutes each time
Computer, Internet			Months	Per week	Minutes
Knitting, Sewing, Needlepoint, Crocheting, Arts and Crafts			Months	Per week	Minutes
Gardening, Landscaping			Months	Per week	Minutes
Snow Shoveling			Months	Per week	Minutes
Maintenance (e.g. car or engine repair), Mechanical Work			Months	Per week	Minutes
Practicing or Playing the Piano			Months	Per week	Minutes
Other Musical Instruments (please specify)			Months	Per week	Minutes
Driving a motorcycle or ATV			Months	Per week	Minutes
Snowmobiling.			Months	Per week	Minutes
Woodworking, furniture building or repair			Months	Per week	Minutes
Remodeling or building a home			Months	Per week	Minutes
Using a chainsaw (e.g. cutting wood) or other vibrating tools			Months	Per week	Minutes
Other (please specify)			Months	Per week	Minutes
Other (please specify)			Months	Per week	Minutes

10. Are you currently:
    a. Pregnant? ____ Yes ____ No
        i. If yes, when is your due date ________/_______/_______
           Month Day Year
    b. Have your periods become irregular or stopped or have you experienced things such as hot flashes? ____ Yes ____ No
        i. If yes, for how many years? ______ Years
        ii. If yes, how long has it been since your last period? ______ _______
            Month Year
    c. Have you used Estrogen replacement (or Hormone Replacement Therapy) ____ Yes ____ No
        i. If yes, how many years have you used Estrogen replacement or Hormone Replacement Therapy? ________ Years
        ii. Did you quit taking Estrogen replacement or Hormone Replacement in the past year? ____ Yes ____ No

11. Have you ever been told by a physician that you have any of the following:

    a. Diabetes: ___ Yes ___ No
       Approximately how many years ago was this diagnosed? _________Years
       With which of the following are you treating the Diabetes?
       ____ Insulin
       ____ Pills / Oral Agents ____
       Both Insulin and Pills ____ Diet
       only (no insulin or pills)
    b. Have you ever been diagnosed with Rheumatoid arthritis, Lupus, or another inflammatory arthritis (not typical Osteoarthritis or Degenerative Arthritis).
       ___ Yes ___ No
       Approximately how many years ago was this diagnosed? _________Years

c. Osteoarthritis or Degenerative Arthritis ___ Yes ___ No

i. If yes, what joints have been affected? (check all that apply)

☑	Body Part	Which side is affected? Right	Left	Both	How many years ago was this diagnosed?
☐	Fingers	☐	☐	☐	Years
☐	Wrists	☐	☐	☐	Years
☐	Elbows	☐	☐	☐	Years
☐	Shoulders	☐	☐	☐	Years
☐	Neck	☐	☐	☐	Years
☐	Back	☐	☐	☐	Years
☐	Knees	☐	☐	☐	Years
☐	Hips	☐	☐	☐	Years
☐	Ankles	☐	☐	☐	Years
☐	Toes	☐	☐	☐	Years

d. Thyroid problem: ___ Yes ___ No
Approximately how many years ago was this diagnosed? _________Years

e. Gout: ___ Yes ___ No
Approximately how many years ago was this diagnosed? _________Years

f. Kidney Failure: ___ Yes ___ No
Approximately how many years ago was this diagnosed? _________Years

g. High Blood Pressure: ___ Yes ___ No
Approximately how many years ago was this diagnosed? _________Years

h. High cholesterol (Laboratory test result over 200 mg/dL) ___ Yes ___ No
Approximately how many years ago was this diagnosed? _________Years

i. Other:___________________________(please specify) ___ Yes ___ No
Approximately how many years ago was this diagnosed? _________Years

j. Other:___________________________(please specify) ___ Yes ___ No
Approximately how many years ago was this diagnosed? _________Years

k. Other:___________________________(please specify) ___ Yes ___ No
Approximately how many years ago was this diagnosed? _________Years

l. Other:___________________________(please specify) ___ Yes ___ No
Approximately how many years ago was this diagnosed? _________Years

m. Other:___________________________(please specify) ___ Yes ___ No
Approximately how many years ago was this diagnosed? _________Years

n. Other:___________________________(please specify) ___ Yes ___ No
Approximately how many years ago was this diagnosed? _________Years

o. Other:___________________________(please specify) ___ Yes ___ No
Approximately how many years ago was this diagnosed? _________Years

p. Other:___________________________(please specify) ___ Yes ___ No
Approximately how many years ago was this diagnosed? _________Years

q. Other:___________________________(please specify) ___ Yes ___ No
Approximately how many years ago was this diagnosed? _________Years

r. Other:___________________________(please specify) ___ Yes ___ No
Approximately how many years ago was this diagnosed? _________Years

12. Has any one in your family (blood relatives only) ever been diagnosed with Carpal Tunnel Syndrome? _____ Yes _____ No

13. What is your height? ______ feet ______ inches

14. What is your current weight? ______ lbs.

15. What is the most you weighed in your life? _______ lbs.

16. What was your weight when you were 20 years old? ________ lbs.

17. Marital Status:
    ____ Never married (Single)
    ____ Currently married
    ____ Divorced
    ____ Separated
    ____ Widowed

18. What is the highest grade in school that you completed?
    ____ 8th grade or less
    ____ Some high school
    ____ High school graduate or GED
    ____ Some college
    ____ College graduate (Bachelor's Degree or higher)

19. How often do you have family problems that irritate or bother you?
    ____ Never
    ____ Occasionally
    ____ Often
    ____ Always

20. Have you ever smoked tobacco?
    ___ Never
    ___ Yes, current
    ___ Yes, but smoked in the past
    If never, go to question 22…otherwise
    How old were you when you started smoking? _____ *years old*
    How old were you when you quit smoking, if you quit?_____ *years old*
    On average, how many cigarettes did/do you smoke per day?______

21. How many cups of caffeinated coffee do you drink in an average day?
    _____ Number of cups per day

22. How many 12 oz. glasses (one can) of caffeinated beverages (e.g. Coke, Pepsi) do you drink in an average day?
    _____ Number of glasses per day

23. Over the past year, how much alcohol do you drink in an average week?
    (1 drink = 12 oz. beer, 6 oz. wine, or 1 oz. liquor)
    _____ None
    _____ 1-2 drinks per week
    _____ 3-5 drinks per week
    _____ 6-11 drinks per week
    _____ 12-17 drinks per week
    _____ 18-23 drinks per week
    _____ 24-29 drinks per week
    _____ 30 or more drinks per week

24. In the past, have you ever had a problem with alcohol? ____ Yes ____ No
   a. If yes, approximately how many years ago? _________ Years

25. How would you describe your general health compared to others of your own age?
____ Much Better
____ Somewhat Better
____ The Same
____ Somewhat Worse
____ Much Worse

26. How often during the past year have you felt "down", blue or depressed?
____ Never
____ Seldom
____ Often
____ Always

27. How often are you physically exhausted after work?
____ Never
____ Seldom
____ Often
____ Always

28. How often are you mentally exhausted after work?
____Never
____ Seldom
____ Often
____ Always

29. Do you get along with your co-workers?
____ Always
____ Often
____ Occasionally
____ Never

30. All in all, how satisfied are you with your job?
____ Very satisfied
____ Satisfied
____ Neither satisfied nor dissatisfied
____ Dissatisfied
____ Very dissatisfied

31. Does your supervisor demonstrate his or her appreciation for the work that you do?
____ Always
____ Often
____ Occasionally
____ Never

32. How strongly would you recommend your job to someone else?
____ Strongly recommend
____ Recommend
____ Neither recommend nor discourage
____ Discourage
____ Strongly discourage

33. If you were looking for a new job now, how likely is it that you would decide to take this job again?
____ Very likely
____ Likely
____ Neither likely nor unlikely
____ Unlikely
____ Very unlikely

34. My employer cares about my health and safety on the job.
____ Strongly agree
____ Agree
____ Neither agree nor disagree
____ Disagree
____ Strongly Disagree

Thank you for completing the questionnaire.

Appendix B.2: Structured Interview

Plant: ______________________

Code # ______________________

Date: ______________________

Baseline Structured Interview (to be completed on a laptop computer)

At the current time or at any time in the past month have you had any pain, ache, stiffness, numbness or tingling in any of the following body parts? (check all that apply and refer to the body diagram so the worker can note all areas that apply

**Body part**	Yes	No	Symptoms in this body part	Pain / Ache / Burning / Stiffness	Pain Severity Rating (1-10)	Current Pain / Ache / Burning / Stiffness Symptoms	Total Pain Duration (days)	Tingling / Numbness	Current Numbness / Tingling Symptoms	Total Numbness / Tingling Duration (days)
1. Neck **(A)**										
				□Yes □No		□Yes □No	Days	□Yes □No	□Yes □No	Days
2. Shoulder										
Interscapular **(D)**			□Yes □No	□Yes □No		□Yes □No	Days	□Yes □No	□Yes □No	Days
R. Nape of the Neck **(C)**			□Yes □No	□Yes □No		□Yes □No	Days	□Yes □No	□Yes □No	Days
R. Periscapular **(G)**			□Yes □No	□Yes □No		□Yes □No	Days	□Yes □No	□Yes □No	Days
R. Shoulder (Glenohumeral) **(H)**			□Yes □No	□Yes □No		□Yes □No	Days	□Yes □No	□Yes □No	Days
R. Upper Arm **(J)**			□Yes □No	□Yes □No		□Yes □No	Days	□Yes □No	□Yes □No	Days
L. Nape of the Neck **(B)**			□Yes □No	□Yes □No		□Yes □No	Days	□Yes □No	□Yes □No	Days
L. Periscapular **(F)**			□Yes □No	□Yes □No		□Yes □No	Days	□Yes □No	□Yes □No	Days
L. Shoulder (Glenohumeral) **(E)**			□Yes □No	□Yes □No		□Yes □No	Days	□Yes □No	□Yes □No	Days
L. Upper Arm **(I)**			□Yes □No	□Yes □No		□Yes □No	Days	□Yes □No	□Yes □No	Days
3. Elbow										
R. Medial Elbow **(N)**			□Yes □No	□Yes □No		□Yes □No	Days	□Yes □No	□Yes □No	Days
R. Lateral Elbow **(P)**			□Yes □No	□Yes □No		□Yes □No	Days	□Yes □No	□Yes □No	Days
R. Elbow (Other) **(O)**			□Yes □No	□Yes □No		□Yes □No	Days	□Yes □No	□Yes □No	Days
R. Forearm **(R)**			□Yes □No	□Yes □No		□Yes □No	Days	□Yes □No	□Yes □No	Days

L. Medial Elbow **(M)**			□Yes □No	□Yes □No		□Yes □No	Days	□Yes □No	□Yes □No	Days
L. Lateral Elbow **(K)**			□Yes □No	□Yes □No		□Yes □No	Days	□Yes □No	□Yes □No	Days
L. Elbow (Other) **(L)**			□Yes □No	□Yes □No		□Yes □No	Days	□Yes □No	□Yes □No	Days
L. Forearm **(Q)**			□Yes □No	□Yes □No		□Yes □No	Days	□Yes □No	□Yes □No	Days
4. Wrist										
R. Wrist **(T)**			□Yes □No	□Yes □No		□Yes □No	Days	□Yes □No	□Yes □No	Days
L. Wrist **(S)**			□Yes □No	□Yes □No		□Yes □No	Days	□Yes □No	□Yes □No	Days
5. Hand										
R. thumb			□Yes □No	□Yes □No		□Yes □No	Days	□Yes □No	□Yes □No	Days
R. index finger			□Yes □No	□Yes □No		□Yes □No	Days	□Yes □No	□Yes □No	Days
R. middle/long finger			□Yes □No	□Yes □No		□Yes □No	Days	□Yes □No	□Yes □No	Days
R. ring finger			□Yes □No	□Yes □No		□Yes □No	Days	□Yes □No	□Yes □No	Days
R. 5th/pinky finger			□Yes □No	□Yes □No		□Yes □No	Days	□Yes □No	□Yes □No	Days
L. thumb			□Yes □No	□Yes □No		□Yes □No	Days	□Yes □No	□Yes □No	Days
L. index finger			□Yes □No	□Yes □No		□Yes □No	Days	□Yes □No	□Yes □No	Days
L. middle/long finger			□Yes □No	□Yes □No		□Yes □No	Days	□Yes □No	□Yes □No	Days
L. ring finger			□Yes □No	□Yes □No		□Yes □No	Days	□Yes □No	□Yes □No	Days
L. 5th/pinky finger			□Yes □No	□Yes □No		□Yes □No	Days	□Yes □No	□Yes □No	Days

1. (Computer selects the body part noted to have been affected in the prior question) In the past month, have you missed work or changed jobs because of problems with the _________body part?

☐ No Missed work, Lost time, or moved to another job.

Body part	Lost time / Missed work	Moved to another job	Number of Days missed work
1. Neck **(A)**	☐Yes ☐No	☐Yes ☐No	Days
2. Shoulder			
Interscapular **(D)**	☐Yes ☐No	☐Yes ☐No	Days
R. Nape of the Neck **(C)**	☐Yes ☐No	☐Yes ☐No	Days
R. Periscapular **(G)**	☐Yes ☐No	☐Yes ☐No	Days
R. Shoulder (Glenohumeral) **(H)**	☐Yes ☐No	☐Yes ☐No	Days
R. Upper Arm **(J)**	☐Yes ☐No	☐Yes ☐No	Days
L. Nape of the Neck **(B)**	☐Yes ☐No	☐Yes ☐No	Days
L. Periscapular **(F)**	☐Yes ☐No	☐Yes ☐No	Days
L. Shoulder (Glenohumeral) **(E)**	☐Yes ☐No	☐Yes ☐No	Days
L. Upper Arm **(I)**	☐Yes ☐No	☐Yes ☐No	Days
3. Elbow			
R. Medial Elbow **(N)**	☐Yes ☐No	☐Yes ☐No	Days
R. Lateral Elbow **(P)**	☐Yes ☐No	☐Yes ☐No	Days
R. Elbow (Other) **(O)**	☐Yes ☐No	☐Yes ☐No	Days
R. Forearm **(R)**	☐Yes ☐No	☐Yes ☐No	Days
L. Medial Elbow **(M)**	☐Yes ☐No	☐Yes ☐No	Days
L. Lateral Elbow **(K)**	☐Yes ☐No	☐Yes ☐No	Days
L. Elbow (Other) **(L)**	☐Yes ☐No	☐Yes ☐No	Days

L. Forearm **(Q)**	☐Yes ☐No	☐Yes ☐No	Days
4. Wrist			
R. Wrist **(T)**	☐Yes ☐No	☐Yes ☐No	Days
L. Wrist **(S)**	☐Yes ☐No	☐Yes ☐No	Days
5. Hand			
R. thumb	☐Yes ☐No	☐Yes ☐No	Days
R. index finger	☐Yes ☐No	☐Yes ☐No	Days
R. middle/long finger	☐Yes ☐No	☐Yes ☐No	Days
R. ring finger	☐Yes ☐No	☐Yes ☐No	Days
R. 5th/pinky finger	☐Yes ☐No	☐Yes ☐No	Days
L. thumb	☐Yes ☐No	☐Yes ☐No	Days
L. index finger	☐Yes ☐No	☐Yes ☐No	Days
L. middle/long finger	☐Yes ☐No	☐Yes ☐No	Days
L. ring finger	☐Yes ☐No	☐Yes ☐No	Days
L. 5th/pinky finger	☐Yes ☐No	☐Yes ☐No	Days

2. (For those indicating a history of numbness OR tingling in the hand/wrist/fingers in question #1):
   How long all together have you had tingling and/or numbness? ___________ months
   Is or was the numbness or tingling in your hands (check all that apply)…
   _____ worse at night
   _____ present on awakening in the morning that resolves within ½ hour
   _____ worse with holding an object (e.g., steering wheel, tool or newspaper)
   _____ Intermittent
   _____ Continuous   If continuous how many months has it been continuous? ________ months

3. Do you ever have numbness and/or tingling in your…
   Right foot/leg? ___ Yes ___ No
   Left foot/leg? ___ Yes ___ No

4. Have you ever had snapping or locking of a finger? _____ Yes _____ No
If yes, Which finger(s)? Right ________________________________ Left __________________________________

5. Have you ever been told by a medical doctor that you have/had any of the following disorders?

Disorder	Yes	No	Right, Left, Bilateral	Year of Diagnosis
Thoracic Outlet Syndrome				
Rotator Cuff Tear				
Rotator Cuff Tendonitis or Shoulder Tendinitis				
Lateral Epicondylitis (Tennis Elbow)				
Medial Epicondylitis (Golfer's Elbow)				
Cubital Tunnel Syndrome (Ulnar nerve problem at the elbow)				
DeQuervains				
Hand / Wrist / Forearm Fracture				
Hand/Wrist Tendonitis				
Carpal Tunnel Syndrome				
Raynaud's Disease				
Trigger Finger				

6. Have you ever had a pinched nerve (e.g. sciatica)? _____ Yes _____ No

If yes, was the pinched nerve in the back? _____ Yes _____ No
Was the pinched nerve in the neck? _____ Yes _____ No

7. Have you ever had a broken bone or fracture? _____ Yes _____ No
If yes, which did you fracture?
R. Clavicle _____ Yes _____ No
L. Clavicle _____ Yes _____ No
R. Humerus _____ Yes _____ No
L. Humerus _____ Yes _____ No

R. Radius _____ Yes _____ No
L. Radius _____ Yes _____ No
R. Ulna _____ Yes _____ No
L. Ulna _____ Yes _____ No
R. Wrist _____ Yes _____ No
L. Wrist _____ Yes _____ No
R. digit(s) _____ Yes _____ No
L. digit(s) _____ Yes _____ No

8. Have you ever dislocated a joint? _____ Yes _____ No

If yes, which joint(s) have you dislocated?
R. Shoulder _____ Yes _____ No
L. Shoulder _____ Yes _____ No
R. Elbow _____ Yes _____ No
L. Elbow _____ Yes _____ No
R. Wrist _____ Yes _____ No
L. Wrist _____ Yes _____ No
Other ____________________________
____
____

9. Have you ever had surgery other than dental, tonsillectomy, C-section or hernia repair? _____ Yes _____ No
If yes, what surgeries?
____
____

10. Have you been hospitalized other than for surgery or childbirth? _____ Yes _____ No
If yes, for what problem(s) or diagnoses?
____
____

Appendix B.3: Sectioned Upper Extremity and Upper Torso Body Diagram

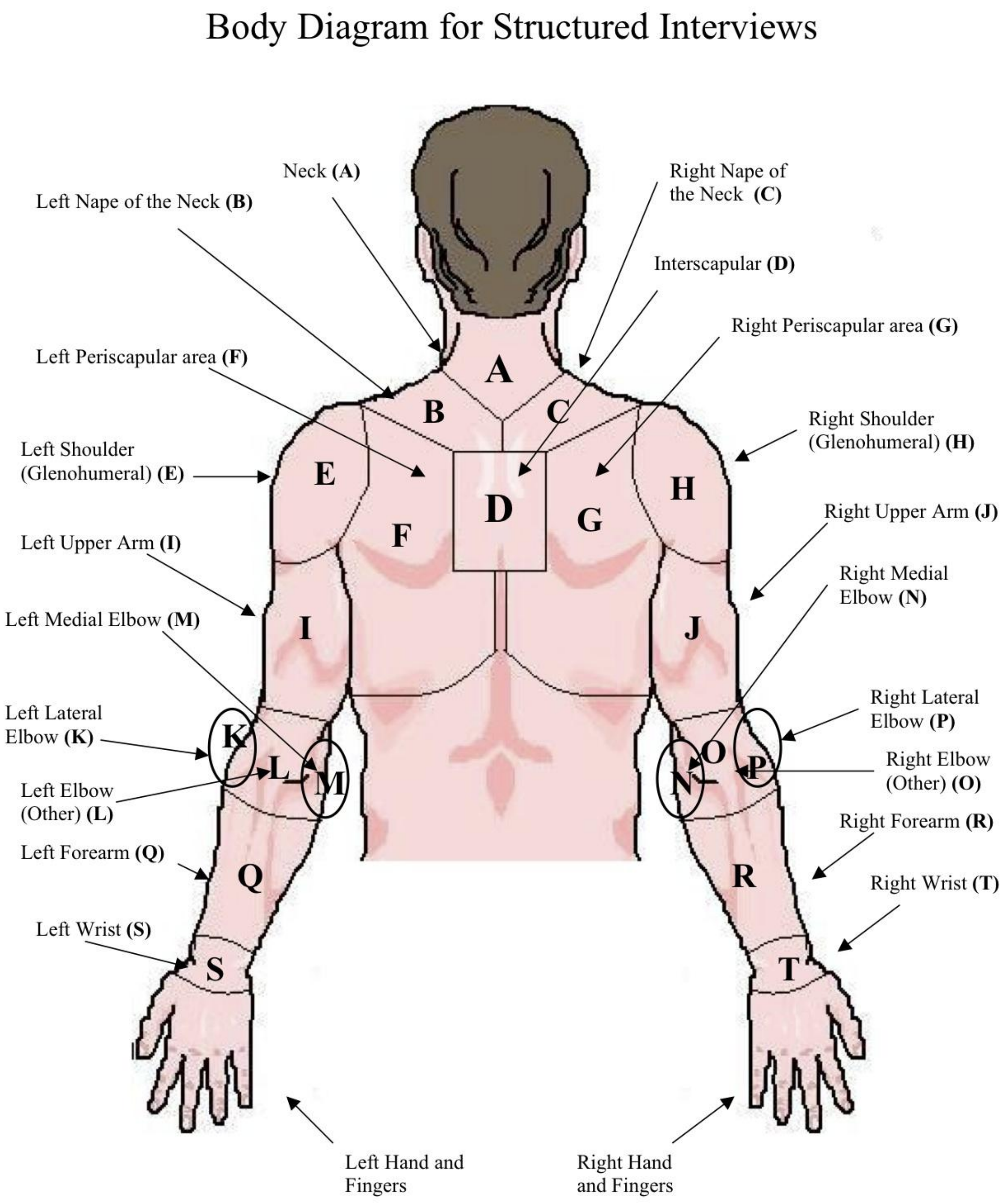

Appendix B.4: Hand and Digit Pain Diagram

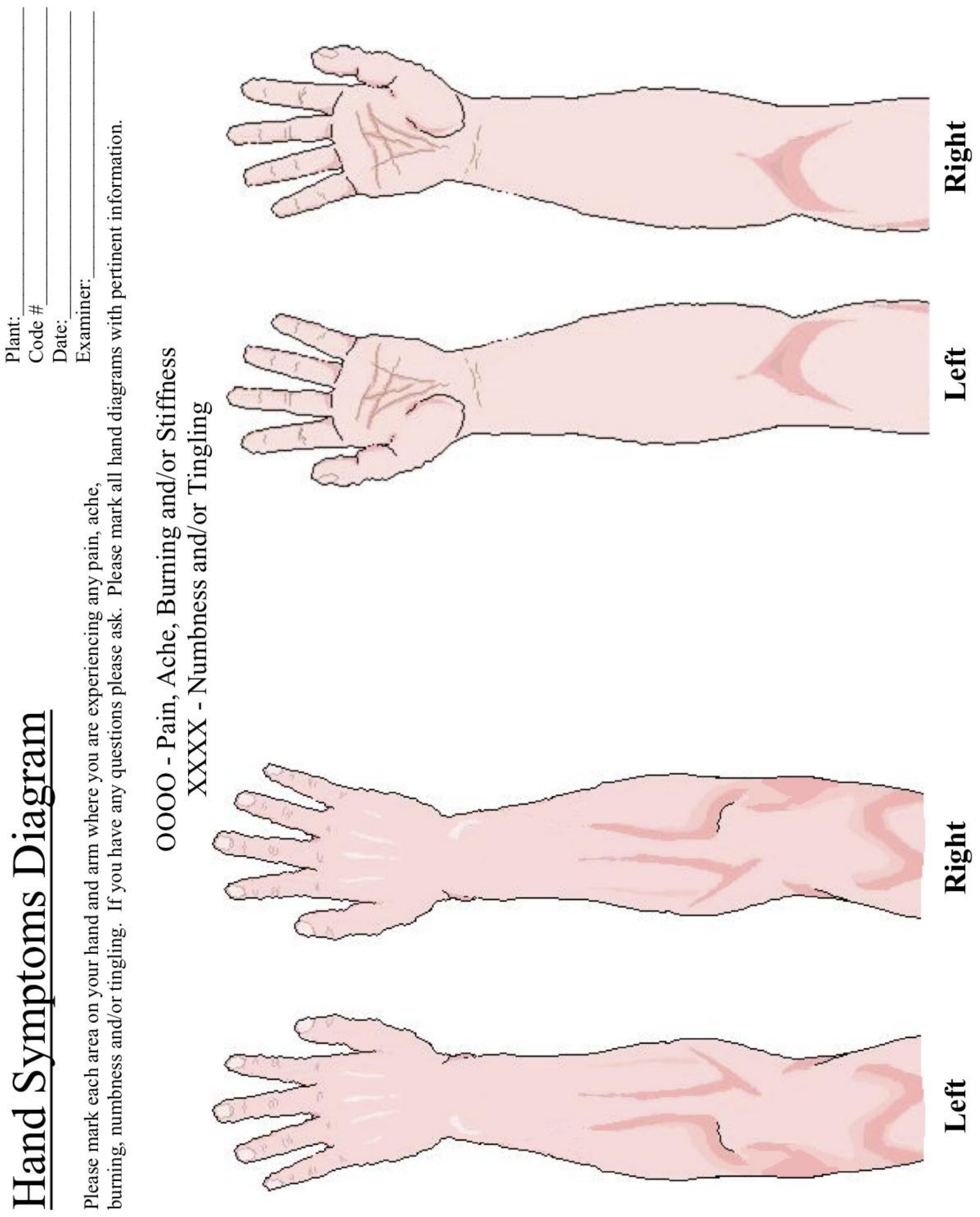

Appendix B.5: Physical Examination Form

Plant: ___________________________
Code # ___________________________
Date: ___________________________

Physical Examination Form (all physicals)

1. Heart Rate _________beats/min
2. Blood Pressure ________mmHg / ________mmHg
   Systolic Diastolic

	Right	_Left_
3. Wrist Depth	____ mm	_____ mm
4. Wrist Width	____ mm	_____ mm
5. Measured Weight	_________kg	
6. Measured Height	__________cm	

Body region	Sign	1st Examiner		2nd Examiner	
		+ Abnormal	- Normal	+ Abnormal	- Normal
Neck	Abnormal Cervical ROM (Flexion less than 2 cm from the sternum)				
	R. Spurlings (Right Neck Rotation)				
	R. Middle Upper Trapezius **"Pain"**				
	R. Levator Scapulae **"Pain"**				
	R. Rhomboid Major **"Pain"**				
	L. Spurlings (Left Neck Rotation)				
	L. Middle Upper Trapezius **"Pain"**				
	L. Levator Scapulae **"Pain"**				
	L. Rhomboid Major **"Pain"**				
R. Shoulder	Painful Arc				
	Shoulder Abduction ROM less than 160 degrees				
	Impingement Sign (Neer)				
	Supraspinatus Test (Empty Can test)				
	External Rotator Weakness				
	Bicipital Tendon Test (Resisted Elbow Flexion)				
L. Shoulder	Painful Arc				
	Shoulder Abduction ROM less than 160 degrees				
	Impingement Sign (Neer)				
	Supraspinatus Test (Empty Can test)				
	External Rotator Weakness				
	Bicipital Tendon Test (Resisted Elbow Flexion)				

1

Plant: __________________ Code # ________________________ Date: _________________________

Region	Sign	1st Examiner + Abnormal	1st Examiner - Normal	2nd Examiner + Abnormal	2nd Examiner - Normal
R. Elbow	Tender Point 1 (Retro Lateral Epicondyle)				
	Tender Point 2 (Lateral Epicondyle)				
	Tender Point 3 (Between Lateral Epicondyle and Radial Head)				
	Tender Point 4 (Radial Head)				
	Tender Point 5 (1 cm Distal to the Radial Head)				
	Tender Point 6 (Radial Tunnel)				
	Tender Medial Epicondyle				
	Tender 1 cm Distal to the Medial Epicondyle				
	Resisted Wrist / Phalangeal Extension (Lateral Epicondylitis pain/soreness/etc.)				
	Lateral epicondyle pain with resisted middle finger extension				
	Resisted Wrist / Phalangeal Flexion (Medial Epicondylitis pain/soreness/etc.)				
	Tinel's Retrocondylar Groove (to Distal Forearm)				
	Tinel's Cubital Tunnel (to Distal Forearm)				
L. Elbow	Tender Point 1 (Retro Lateral Epicondyle)				
	Tender Point 2 (Lateral Epicondyle)				
	Tender Point 3 (Between Lateral Epicondyle and Radial Head)				
	Tender Point 4 (Radial Head)				
	Tender Point 5 (1 cm Distal to the Radial Head)				
	Tender Point 6 (Radial Tunnel)				
	Tender Medial Epicondyle				
	Tender 1 cm Distal to the Medial Epicondyle				
	Resisted Wrist / Phalangeal Extension (Lateral Epicondylitis pain/soreness/etc.)				
	Lateral epicondyle pain with resisted middle finger extension				
	Resisted Wrist / Phalangeal Flexion (Medial Epicondylitis pain/soreness/etc.)				
	Tinel's Retrocondylar Groove (to Distal Forearm)				
	Tinel's Cubital Tunnel (to Distal Forearm)				

Mark All Positive Findings

Mark All Positive Findings

2

Plant: ___________________ Code # _________________________ Date: __________________________

Region	Sign	1st Examiner + Abnormal	1st Examiner - Normal	2nd Examiner + Abnormal	2nd Examiner - Normal
R. Wrist	FCR – tenderness				
	FCU – tenderness				
	Flexor tendon- tenderness				
	1st compartment tenderness				
	Tender over Extensor Compartment (not 1st compartment)				
	If yes, which Compartment?				
	ECU tenderness				
	Resisted wrist flexion with pain at FCR				
	Resisted wrist flexion with pain at FCU				
	Resisted Phalangeal Flexion				
	Finkelstein's (1st Ext. Comp. pain/soreness/etc.)				
	Pain in Extensor Tendon with resisted Phalangeal extension (not 1st compartment)				
	Pain in ECU with resisted extension				
	Phalen's 60 second test ($\geq$ 2 median nerve digits)				
	Tinel's Proximal Carpal Tunnel ($\geq$ 2 median nerve digits)				
	Tinel's Mid-Carpal Tunnel ($\geq$ 2 median nerve digits)				
	Tinel's Distal Carpal Tunnel ($\geq$ 2 median nerve digits)				
L. Wrist	FCR – tenderness				
	FCU – tenderness				
	Flexor tendon- tenderness				
	1st compartment tenderness				
	Tender over Extensor Compartment (not 1st compartment)				
	If yes, which Compartment?				
	ECU tenderness				
	Resisted wrist flexion with pain at FCR				
	Resisted wrist flexion with pain at FCU				
	Resisted Phalangeal Flexion				
	Finkelstein's (1st Ext. Comp. pain/soreness/etc.)				
	Pain in Extensor Tendon with resisted Phalangeal extension (not 1st compartment)				
	Pain in ECU with resisted extension				
	Phalen's 60 second test ($\geq$ 2 median nerve digits)				
	Tinel's Proximal Carpal Tunnel ($\geq$ 2 median nerve digits)				
	Tinel's Mid-Carpal Tunnel ($\geq$ 2 median nerve digits)				
	Tinel's Distal Carpal Tunnel ($\geq$ 2 median nerve digits)				

Plant: ___________________ Code # ________________________ Date: _________________________

Region	Sign	1st Examiner		2nd Examiner	
		+ Abnormal	- Normal	+ Abnormal	- Normal
R. Fingers	Thumb – A1 tenderness				
	Tendon Nodule				
	Locking / Triggering				
	Index – A1 tenderness				
	Tendon Nodule				
	Locking / Triggering				
	Middle finger – A1 tenderness				
	Tendon Nodule				
	Locking / Triggering				
	Ring finger – A1 tenderness				
	Tendon Nodule				
	Locking / Triggering				
	5th digit – A1 tenderness				
	Tendon Nodule				
	Locking / Triggering				
	CMC Deformity				
	CMC Grind Test				
L. Fingers	Thumb – A1 tenderness				
	Tendon Nodule				
	Locking / Triggering				
	Index – A1 tenderness				
	Tendon Nodule				
	Locking / Triggering				
	Middle finger – A1 tenderness				
	Tendon Nodule				
	Locking / Triggering				
	Ring finger – A1 tenderness				
	Tendon Nodule				
	Locking / Triggering				
	5th digit – A1 tenderness				
	Tendon Nodule				
	Locking / Triggering				
	CMC Deformity				
	CMC Grind Test				

Plant: __________________ Code # ________________________ Date: _________________________

7. Signs of Rheumatoid Arthritis ____ Yes ____ No
8. Heberden's Nodes ____ Yes ____ No
   If yes, which joint(s)___________________________________
9. Bouchards Nodes ____ Yes ____ No
   If yes, which joint(s)___________________________________

	**Right**	**Left**
10. Dorsal Wrist Ganglia	___ Yes ___ No	___Yes ___ No
11. Volar Wrist Ganglia	___ Yes ___ No	___Yes ___ No
12. Dupuytren's contracture	___ Yes ___ No	___Yes ___ No

Other findings in the physical exam:

Body Part	Test performed	Positive finding	Negative finding	Examiner	
				1st	2nd

Current Musculoskeletal Disorder(s)

Right	Left	Diagnostic Impression

Prior/Past Musculoskeletal Disorder(s)

Right	Left	Diagnostic Impression

5

Appendix B.6: Nerve Conduction Form

Nerve Conduction Data Form

Plant:____________________________
Subject ID #______________________
Date:_____________________________
Examiner:_________________________

Right Hand		
**Transcarpal**	**Digital Sensory**	**Motor**
Distance ___ 8 cm ___ ( )	Distance _____14 cm ___( )	Distance ____ 8 cm ____ ( )
Median Latency (msec)	Median Latency (msec)	Median Latency (msec)
Median Amplitude (μVs)	Median Amplitude (μVs)	Median Amplitude (K)
Ulnar Latency (msec)	Ulnar Latency (msec)	Ulnar Latency (msec)
Ulnar Amplitude (μVs)	Ulnar Amplitude (μVs)	Ulnar Amplitude (K)
Comments	Comments:	Comments

Left Hand		
**Transcarpal**	**Digital Sensory**	**Motor**
Distance ___ 8 cm ___ ( )	Distance _____14 cm ____( )	Distance ____ 8 cm ____ ( )
Median Latency (msec)	Median Latency (msec)	Median Latency (msec)
Median Amplitude (μVs)	Median Amplitude (μVs)	Median Amplitude (K)
Ulnar Latency (msec)	Ulnar Latency (msec)	Ulnar Latency (msec)
Ulnar Amplitude (μVs)	Ulnar Amplitude (μVs)	Ulnar Amplitude (K)
Comments	Comments	Comments

Hand Dorsum Temperature = 32° C
Median Digital Sensory → Long Finger   Ulnar Digital Sensory → Little Finger
If transcarpal delta ≥ 0.4 msec, do ulnat digital sensory and ulnar motor
If median motor latency or amplitude abnormal, map E1.   If still abnormal, do median/ulnar with forearm conductions
If mapping needed for motor studies, note both original and mapped numbers in comments.

Appendix B.7: Pain Scale

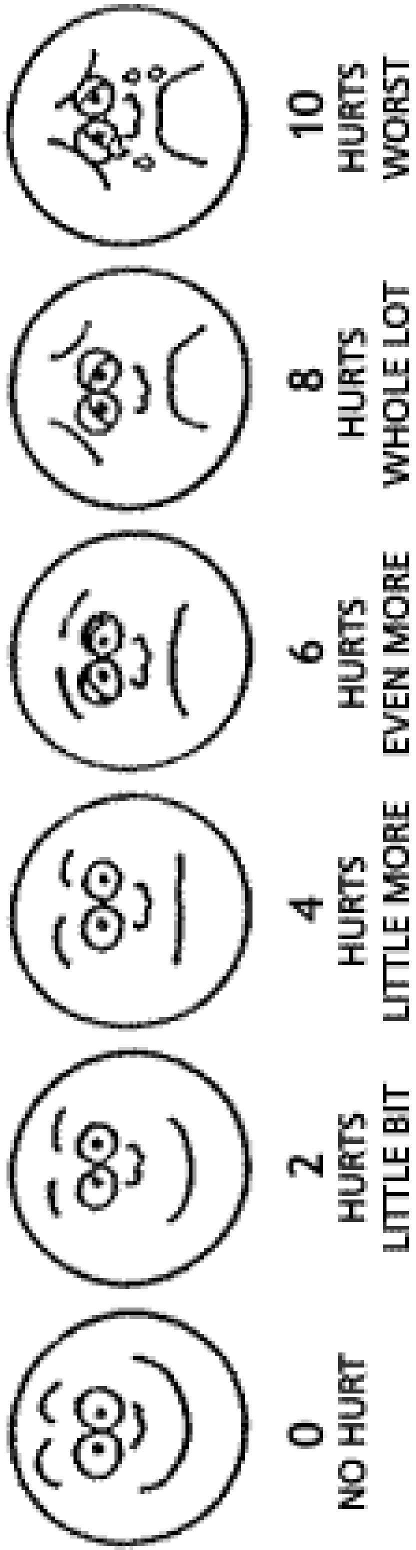

Supplement: Additional file 1 — Appendix B: Health Outcomes Data Collection Instruments. [file 1471-2474-13-90-S1.pdf]
