# Supplementary material for: The WISTAH hand study: A prospective cohort study of distal upper extremity musculoskeletal disorders
Source: BMC Musculoskelet Disord. 2012 Jun 6;13:90. doi: 10.1186/1471-2474-13-90 (PMC3476983; doi:10.1186/1471-2474-13-90)

Appendix C:

Job Physical Exposure Data Collection Instruments

C.1 Position Specific Data Collection Form ....

C.2 Job Specific Data Collection Form ....

C.3 Borg CR-10 Scale ....

C.4 Grip/Pinch Diagram ....

C.5 Hand Activity Level (HAL) Scale ....

Appendix C.1: Position Specific Data Form

Position / Worker Specific Data Form

1. Subject I.D. ______________________ <AA0001>
5. Facility: ______________________

2. Subject Name: ______________________
6. Time (24hr): ______________________ <2:30 pm = 14:30, midnight = 00:00>

3. ☐Male ☐Female
4. Age: ________ <years>
7. Date: ______________________ <MM / DD / YYYY>

8. Analyst #1: ______________________
9. Analyst #2: ______________________

Position Information

10. Line / Department Title: ______________________

11. Position Title: ______________________

12. Position Description: ______________________

____

____

____

13. Typical Shift ***Start*** Time (24hr): ________

14. Typical Shift ***End*** Time (24hr): ________

15. Break-Time (Minutes/day): ________ Minutes <Total of Lunch & Breaks / Day>

16. # of ***Distinct*** Jobs Rotated to: ________

17.

☐ M	☐ T	☐ W	☐ H	☐ F	☐ S	☐ SU	Typical ODD WEEK <Check Days Worked, Write Total Hours/Day Below>
__	__	__	__	__	__	__	

☐ M	☐ T	☐ W	☐ H	☐ F	☐ S	☐ SU	Typical EVEN WEEK <Check Days Worked, Write Total Hours/Day Below>
__	__	__	__	__	__	__	

18. Note Unusual Schedule Here: ______________________

____

19. Jobs Included in Position:

Job #	Line/Cell/ Workstation	Job Title/ Description	PACE Self, Line, Piece Rate	Cycle Time (seconds)	Production per Hour	Typical Work Hrs/Day (OR)	Typical Work % of Day
1			S L P				
2			S L P				
3			S L P				
4			S L P				
5			S L P				
6			S L P				
7			S L P				
8			S L P				

1

Subject ID: ____________________________

Worker Information

20. Prior Work Experience; Back Maximum of 10 Years ***OR*** Maximum of 5 Jobs[1]:
(Include Current Position and *significantly* different prior positions with *present* employer first)

Position	Title / Description	Years	Average Borg Rating
Current			
Prior #1			
Prior #2			
Prior #3			
Prior #4			
Prior #5			

21. Do you currently work on a second job outside of this facility? Yes No

22. If Yes, 2nd Job Title/Description: ____

____

23. Average Hours/Week on 2nd Job: _________ 24. Number of Years on 2nd Job: ___________

25. Dominant Hand Overall (Average) Borg Rating for 2nd Job: __________________________

	Trial #1	Trial #2	Trial #3	Average
26. Worker's Dominant Hand L R B (if Both, Test Right)				
27. Worker's Maximum ***Grip*** Strength (Dominant Hand, #2 Position)	kgf	kgf	kgf	kgf
28. Worker's Maximum ***Lateral Pinch*** Strength (Dominant Hand)	kgf	kgf	kgf	kgf
29. Worker's Maximum ***3-Point Pinch*** Strength (Dominant Hand)	kgf	kgf	kgf	kgf
30. Standardized Grip Force[2] (10 kgf) (Dominant Hand, Borg CR-10)	Borg CR-10			

	Worker Estimated Rating (Stress) (Dominant Hand)	Worker Rating @ Beg/End (Stress) (Dominant Hand)
31. Overall Worker Rating at ***Beginning*** of Shift[3] (Borg CR-10)		
32. Overall Worker Rating at ***End*** of Shift[3] (Borg CR-10)		

33. Analyst Notes (Optional): ____

____

____

____

Appendix C.2: Job Specific Data Collection Form

Job Specific Data Form

1. Subject I.D. ______________________________
   <From Position Form, Field #1>
2. Analyst Name(s): ________________________
3. Cycle Time (minutes:seconds): ________________
4. Job ID (Plant Official):________________________
5. Facility: ________________________________
6. Time (24hr): ____________________________
   <2:30 pm = 14:30, midnight = 00:00>
7. Date: ____________________________________
   <MM / DD / YYYY>
8. Job # (From “Position Data Form, Field #19”):________

Job Overview

9. Task Borg CR-10 Estimated Ratings (To Assist with Video Analysis)*

Task	Task Description	Average Analyst Borg CR-10 Rating	
		Left *(Force)*	*Right* *(Force)*
1			
2			
3			
4			
5			

* Write down major tasks under “Task Description”, assign typical Borg CR-10 ratings for hand(s) involved for each task

Video Observation (Remind worker: “I will focus on your hands & arms.”)

Direct Observation (Worker Remains Working.)

	Left Hand			Right Hand		
10. Average Hand/Arm Vibration Exposure, (Circle Level(s) if Present, Indicate % of Cycle Time for ***EACH*** Level)	Neg.	Visible	Severe	Neg.	Visible	Severe
	_____%	______%	______%	_____%	______%	______%

	Left Hand		Right Hand	
11. Gloves (Indicate Type & Fit for ***BOTH*** Hands)	☐ *NONE*<br>☐ Vinyl<br>☐ Latex<br>☐ Cotton<br>☐ Tipless<br>☐ Cut-Resistant<br>☐ Anti-Vibration<br>☐ Leather<br>☐ Other______	☐ Tight<br>☐ Normal<br>☐ Loose	☐ *NONE*<br>☐ Vinyl<br>☐ Latex<br>☐ Cotton<br>☐ Tipless<br>☐ Cut Resistant<br>☐ Anti-Vibration<br>☐ Leather<br>☐ Other______	☐ Tight<br>☐ Normal<br>☐ Loose

12. Room Temperature:______ºC	Left Hand	Right Hand
13. Hand Contact with Hot/Cold Objects (Indicate Temperature, % of Cycle Time and Use of Gloves for ***BOTH*** Hands)	____ºC ☐ *Not Applicable*<br>Gloves: ☐ Yes ☐ No	____ºC ☐ *Not Applicable*<br>Gloves: ☐ Yes ☐ No
	____% of Cycle in Contact	____% of Cycle in Contact

Job #____-1

Form #: 12082003

Subject I.D. ______________________________ Job # (From “Personal Data Form, Field #19”): __________

14. Localized Mechanical Compression (Determine Severity on Site):

	Left: Negligible	Left: Moderate / Severe	Left: Exertions/cycle % of Cycle	Right: Negligible	Right: Moderate / Severe	Right: Exertions/cycle % of Cycle
a. Palm		M S	Ex/Cycle:____ % of Cycle:_____		M S	Ex/Cycle:____ % of Cycle:_____
b. Wrist		M S	Ex/Cycle:____ % of Cycle:_____		M S	Ex/Cycle:____ % of Cycle:_____
c. Forearm		M S	Ex/Cycle:____ % of Cycle:_____		M S	Ex/Cycle:____ % of Cycle:_____
d. Elbow		M S	Ex/Cycle:____ % of Cycle:_____		M S	Ex/Cycle:____ % of Cycle:_____
e. Finger(s)		M S	Ex/Cycle:____ % of Cycle:_____		M S	Ex/Cycle:____ % of Cycle:_____

Worker Ratings (Final Interaction with Worker for this Job)

15. Hand / Wrist / Forearm / Elbow Force Measures:

Variables	Not Applicable	Typical Overall Exposure: *Left*	Typical Overall Exposure: *Right*	Typical Peak Exposure: *Left*	Typical Peak Exposure: *Right*
a. Weight of Workpiece(s) or tool(s) <kg> (supported by worker)		kg	kg	kg	kg
b. Center of Mass Offset, <inches> (Measure from Center of Grip)		in	in	in	in
c. Matching Grip Force[4], <kgf> (Dominant Hand Only)		kgf	kgf	kgf	kgf
d. Matching Pinch Force[5], <kgf> Typical Pinch Type: ☐ Lateral, ☐ 2-Point, ☐ 3-Point		kgf	kgf	kgf	kgf
e. Matching Thrust Force[6], <kgf>		kgf	kgf	kgf	kgf
f. Pushing Force, <kgf> (Analyst Measured)		kgf	kgf	kgf	kgf
g. Pulling Force, <kgf> (Analyst Measured)		kgf	kgf	kgf	kgf
h. Analyst Rating of Applied Force[7,*] (Borg CR-10, Entire Job)		CR-10	CR-10	CR-10	CR-10
i. Worker Rating of Applied Force[7,*] (Borg CR-10, Entire Job)		CR-10	CR-10	CR-10	CR-10

* Typical Stress Level Across ***ALL*** Sub-Tasks for **One** Cycle

Job #____-2

Form #: 12082003

Subject I.D. ______________________________ Job # (From "Personal Data Form, Field #19"): __________

Analysis from Video (Performed outside the plant):

16. Tool Kicks and Hand as Hammer:

	Left: Negligible	Left: Number/Cycle @ Each Severity Level – Moderate (Visible)	Left: Severe	Right: Negligible	Right: Number/Cycle @ Each Severity Level – Moderate (Visible)	Right: Severe
a. Tool Kicks						
b. Hand as Hammer						

17. HAL Rating, Hand/Wrist Posture, Forearm Rotations (All Measurements Taken from Video)	Typical Exposure %		Typical Exposure Counts (Circle Peak Force Posture)	
	Left Hand	Right Hand	Left Hand	Right Hand
a. HAL Rating				
b. Hand/Wrist Posture (From ***Anatomical*** Neutral, Measured in degrees)				
I. Flexion (Totals 100% of Cycle) — Low <30º, Med 30º-50º, Hi >50º	L M H<br>___ ___ ___	L M H<br>___ ___ ___	L M H<br>___ ___ ___	L M H<br>___ ___ ___
II. Extension (Totals 100% of Cycle) — Low <30º, Med 30º-50º, Hi >50º	L M H<br>___ ___ ___	L M H<br>___ ___ ___	L M H<br>___ ___ ___	L M H<br>___ ___ ___
III. Ulnar Deviation (Totals 100% of Cycle) — Low <10º, Med 10º-25º, Hi >25º	L M H<br>___ ___ ___	L M H<br>___ ___ ___	L M H<br>___ ___ ___	L M H<br>___ ___ ___
IV. Radial Deviation (Totals 100% of Cycle) — Low <5º, Hi ≥5º	L H<br>____ ____	L H<br>____ ____	L H<br>____ ____	L H<br>____ ____
c. Number of Forearm Rotations per Cycle (Measure rotations ≥ ±45º, return to neutral is 1 rotation)				

18. Exertions with Elbow Included Angle <70º or >135º

(Record exertions / cycle, % of cycle, and typical forearm rotation during exertion):

	Left: Negligible	Left: <70º	Left: >135º	Right: Negligible	Right: <70º	Right: >135º
a. Number of Exertions		_____ / Cycle	_____ / Cycle		_____ / Cycle	_____ / Cycle
b. % of Cycle Time		_________ %	_________ %		_________ %	_________ %
c. Typical Forearm Position (**N**eutral, **P**rone, **S**upine)		N ☐ P ☐ S ☐	N ☐ P ☐ S ☐		N ☐ P ☐ S ☐	N ☐ P ☐ S ☐

Job #____-3

Form #: 12082003

Subject I.D. ______________________________ Job # (From "Personal Data Form, Field #19"): __________

19. Grip & Pinch Exertions for a Typical Cycle (Record % of cycle, and Grip or Pinch Span (≈ inches)):

Type of Grasp (Indicate: % of Cycle & Grip or Pinch Span)	Left			Right		
	Negligible	% of Cycle	≈ Grip/Pinch Span*	Negligible	% of Cycle	≈ Grip/Pinch Span*
a. Power/Hook Grip			inches			inches
b. Oblique Grip			inches			inches
c. Palmer Grip			inches			inches
d. Palmer Pinch			inches			inches
e. 3-Point Pinch			inches			inches
f. 2-Point Pinch			inches			inches
g. Lateral (Key) Pinch			inches			inches
h. 2-Finger "Scissor" Pinch			inches			inches

* Approximate actual span from video observation; for spans less than ~¼ inch, put 0 for Grip/Pinch Span.

Strain Index Analysis from Video:

20. Total Cycle Time = ________Seconds (As Timed at Plant, or From Video)

21. ***LEFT*** Hand Strain Index Table:

Task	Task Description	Time	Intensity of Exertion (Borg CR-10)	Number of Exertions / **Cycle**	Hand/Wrist Posture (SI Definition)	**Duration** of Exertion per **Cycle**	Speed (SI Definition)	Hours / Day
1								
2								
3								
4								
5								
6								
7								
8								
9								
10								
11								
12								
13								
14								
15								

Subject I.D. ______________________________ Job # (From "Personal Data Form, Field #19"): __________

22. ***RIGHT*** Hand Strain Index Table:

Task	Task Description	Time	Intensity of Exertion (Borg CR-10)	Number of Exertions / **Cycle**	Hand/Wrist Posture (SI Definition)	**Duration** of Exertion per **Cycle**	Speed (SI Definition)	Hours / Day
1								
2								
3								
4								
5								
6								
7								
8								
9								
10								
11								
12								
13								
14								
15								

23. ***Comments & Observations (Risk Factors and Concerns not Otherwise Recorded)***:

Appendix C.3: Borg CR-10 Scale

Borg CR-10

**0.5**	**Very, Very Light**
**1**	**Very Light**
**2**	**Light**
**3**	**Moderate**
**4**	**Somewhat Hard**
**5**	**Hard**
**6**	
**7**	**Very Hard**
**8**	
**9**	
**10**	**Near Maximal**

Appendix C.4: Grip/Pinch Diagram

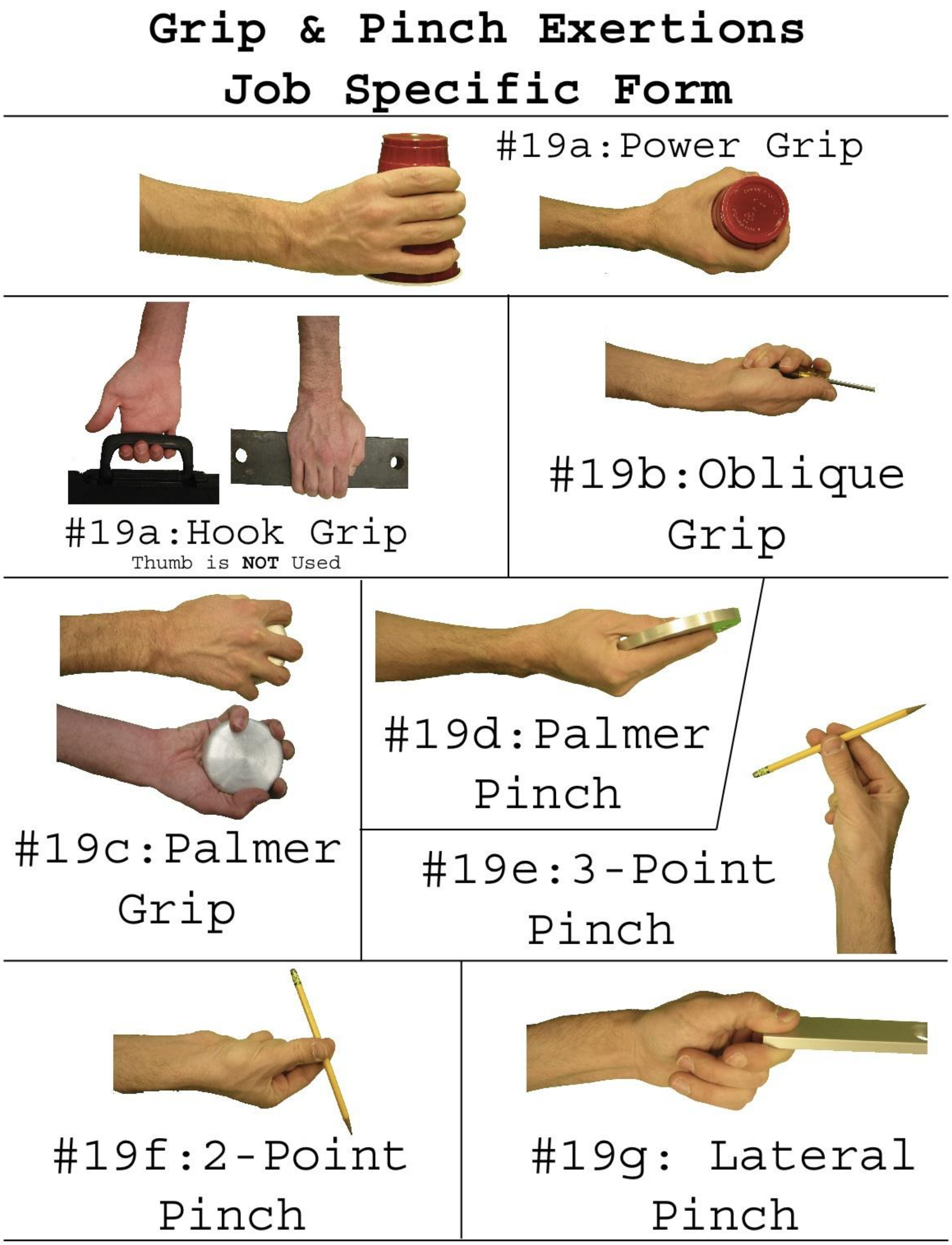

Grip & Pinch Strength Testing
Position Specific Form

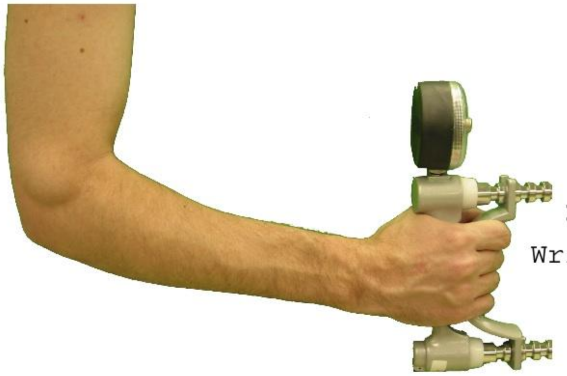

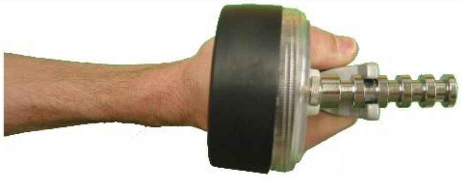

#27 Power Grip

Wrist Posture: Functional Neutral
Shoulder Angle: 0°
Elbow Angle: 90°

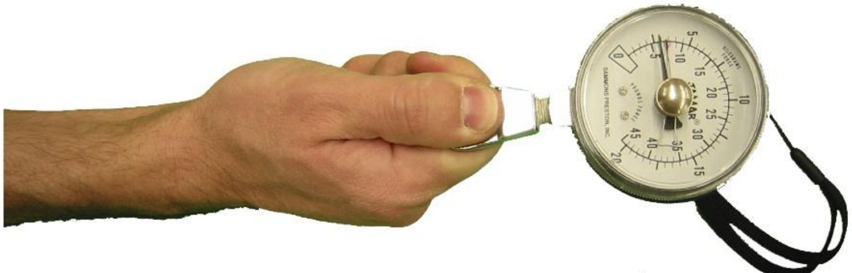

#28: Lateral Pinch

Wrist Posture: Functional Neutral
Use: Thumb, Side of Index Finger

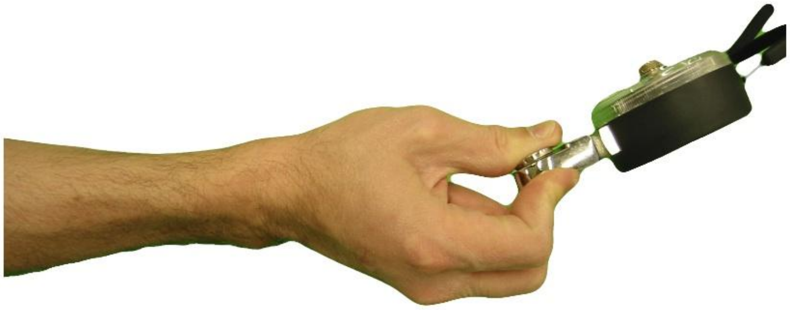

#29: 3-Point Pinch

Wrist Posture: Functional Neutral
Use: Thumb, Index & Middle Fingers

Appendix C.5: Hand Activity Level (HAL) Scale

HAL Scale

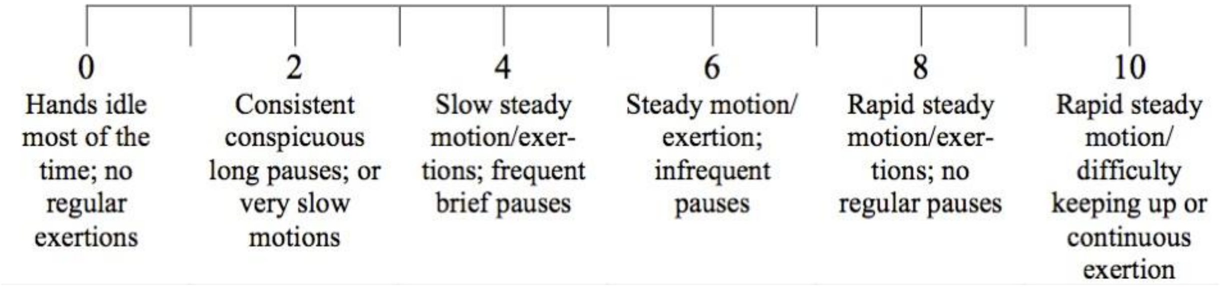

Supplement: Additional file 2 — Appendix C: Job Physical Exposure Data Collection Instruments. [file 1471-2474-13-90-S2.pdf]
