# Supplementary material for: The WISTAH hand study: A prospective cohort study of distal upper extremity musculoskeletal disorders
Source: BMC Musculoskelet Disord. 2012 Jun 6;13:90. doi: 10.1186/1471-2474-13-90 (PMC3476983; doi:10.1186/1471-2474-13-90)
Supplement: Additional file 3 — Appendix A: Job Classification (PDF 215 kb) [file 1471-2474-13-90-S3.pdf]

Appendix A:

A.1 Initial Classification of Jobs into Low, Medium and High Exposures..........
A.2 Field Data Collection Instructions ....

Appendix A.1: Initial Classification of Jobs into Low, Medium, and High Exposure

JOB EVALUATION for INITIAL CLASSIFICATION of JOBS INTO LOW, MEDIUM and HIGH EXPOSURE CATEGORIES

Date ________________________ Analyst ____________________________

1. Name ______________________ 2. Plant ______________________________

3. Department _________________ 4. Job Title ___________________________

5. Sub-Job Title ________________ 6. Shift _______________________________

7. Does worker rotate to another job? � Yes � No

If Yes, jobs the observed worker rotates to:

a. Job Title _______________ Department________________
_______________________ Hours/Shift___________________

b. Job Title _______________ Department________________
_______________________ Hours/Shift___________________

c. Job Title _______________ Department________________
_______________________ Hours/Shift___________________

d. Job Title _______________ Department________________
_______________________ Hours/Shift___________________

8. Cycle time (O/V)_______________________________________seconds

9. Duration of exposure on this job (O) _____________________hours/day

10. Length of shift (O) ____________________________________hours/day

**Force (Borg Rating)**	**Number of Exertions per Minute**	**% Duration of Exertion**	**Hand/Wrist Posture Flexion/Extension**
≤ 2 � L<br>3-4 � M<br>≥ 5 � H	≤ 4 � L<br>5-8 � M<br>> 8 � H	≤ 20% � L<br>21-40% � M<br>> 40% � H	≤ 20º � G<br>21-40º � F<br>> 40º � P

Table A-1: Combinations of Force, Repetition, % Duration of Exertion and Hand/Wrist Posture to be Used to Initially Classify Jobs into High, Medium and Low Risk Groups.

High Risk				Medium Risk				Low Risk			
**F**	**R**	**D**	**P**	**F**	**R**	**D**	**P**	**F**	**R**	**D**	**P**
H	L	M	P	H	L	L	P	H	L	L	G
H	L	H	G	H	L	M	G	H	L	L	F
H	L	H	F	H	L	M	F	M	L	L	G
H	L	H	P	H	M	L	G	M	L	L	F
H	M	L	P	H	M	L	F	M	L	L	P
H	M	M	G	M	L	M	P	M	L	M	G
H	M	M	F	M	L	H	G	M	L	M	F
H	M	M	P	M	L	H	F	M	M	L	G
H	M	H	G	M	M	L	P	M	M	L	F
H	M	H	F	M	M	M	G	L	L	L	G
H	M	H	P	M	M	M	F	L	L	L	F
H	H	L	G	M	H	L	G	L	L	L	P
H	H	L	F	M	H	L	F	L	L	M	G
H	H	L	P	M	H	L	P	L	L	M	F
H	H	M	G	M	H	M	G	L	L	M	P
H	H	M	F	M	H	M	F	L	L	H	G
H	H	M	P	M	H	M	P	L	L	H	F
H	H	H	G	L	L	H	P	L	M	L	G
H	H	H	F	L	M	H	G	L	M	L	F
H	H	H	P	L	M	H	F	L	M	L	P
M	L	H	P	L	M	H	P	L	M	M	G
M	M	M	P	L	H	L	P	L	M	M	F
M	M	H	G	L	H	M	G	L	M	M	P
M	M	H	F	L	H	M	F	L	H	L	G
M	M	H	P	L	H	M	P	L	H	L	F
M	H	H	G	L	H	H	G				
M	H	H	F	L	H	H	F				
M	H	H	P								
L	H	H	P								

F = Force (H = high, M = medium, L = low)
R = Repetition (H = high, M = medium, L = low)
D = % Duration of Exertion (H = high, M = medium, L = low)
P = Hand/Wrist Posture (G = good, F = Fair, P = poor)

Appendix A.2: Field Data Collection Instructions

Position / Worker Specific Data Form Instructions

1. Prior Work Experience Starting From Current Job

a. **I will ask you the overall or average level of stress you feel on your arm while performing your present job as well as performing previous jobs. (Remember, "arm" is from your elbow to fingertips).**
b. **Concentrate on your dominant arm.**
c. **Please rate the overall or average level of physical stress you feel on your arm for your current job including all rotations.**
d. **What job were you performing before this job?**
e. **Please rate the overall or average level of physical stress on you arm for your previous job(s).**
f. **Follow steps d and e for other previous jobs up to a total of** 5 previous jobs or 10 years of employment**.**

2. Standardized Grip Force (10Kg), Dominant Hand

a. **Please grip this device and** slowly **increase your force until the pointer is in the red area. (*Don and Richard will modify grip dynamometers so that there is a pointer and red area in the back of the gauge corresponding to 10 Kg.*) Keep holding it until I ask you to relax.**
b. **Concentrate on your dominant arm. (Remember, arm is hand/wrist/forearm/elbow). I will ask you to rate the level of physical stress you feel in your dominant arm.**
c. **Let the worker apply force for 3-4 seconds.**
d. **Please rate the level of physical stress on your arm.**

3. Beginning and End of Shift Ratings

a. **Some people feel the same stress on their hand/wrist/forearm/elbow throughout the shift. Others feel different levels of physical stress at the beginning and end of a shift (job rotation included).**
b. **Concentrate on your dominant arm.**
c. **Please rate the overall or average level of physical stress on your arm at the beginning of your work shift on a typical workday. (Remember, "arm" is from your elbow to your fingertips.)**
d. **Please rate the overall or average level of physical stress on your arm at the end of your work shift on a typical workday. (Remember, "arm" is from your elbow to your fingertips.)**

4. Matching Grip Force

    1. **When you use this (name of the tool or work piece), you apply a certain amount of pressure to hold it while using it.**
    2. **Please hold this device (dynamometer) and apply the same amount of pressure that you apply when holding the hand tool/work piece.**

5. Matching Pinch Force
   Select the Type of Pinch Used by the Worker and Note it Down
   (Lateral, 2 point, 3 point, Use 3 point for Palmer)

    1. **When you use this (name of the tool or work piece) or perform this task, you apply a certain amount of pressure to hold the (tool or work piece) while using it.**
    2. **Please hold this device (pinch meter) and apply the same amount of pressure that you apply when holding the (tool or work piece).**

6. Matching Thrust Force

    1. **When you use this (name of tool), you apply a certain amount of pressure to push the tool while using it. (Often it will be pushing down but it could be horizontal).**
    2. **Please hold this device and push it with the same amount of pressure that you use when pushing your hand tool.**

7. Instructions For Borg Scale-Estimating Force for the Job

    1. **Think about performing your job just** ONE **time or for** ONE **exertion.** DO NOT **think about getting tired from doing your job for your entire shift.**
    2. **If you were to produce one (part/unit/perform one cycle) then rate the average or overall level of physical stress you feel on your RIGHT arm.**
    3. **If you were to produce one (part/unit/perform one cycle) then rate the maximum level of physical stress you feel on your RIGHT arm.**
    4. **If you were to produce one (part/unit/perform one cycle) then rate the average or overall level of physical stress you feel on your LEFT arm.**
    5. **If you were to produce one (part/unit/perform one cycle) then rate the maximum level of physical stress you feel on your LEFT arm.**

General Instructions

1. Using this rating scale *(place the Borg CR-10 Scale in front of the worker)*, I will ask you to rate the level of physical stress you feel while performing your job.
2. Choose the words that best describe the level of physical stressyou feel.
3. Do this for both your right arm and your left arm separately.
4. For all my questions please concentrate on your hand/wrist/forearm/elbow. This is the area between your elbowand your fingertips. We will refer to this area as “arm”.
5. Concentrating only on this area, rate the level of physical stress and where you feel it the most (for example: I feel the most stress in the wrist).
6. DO NOT think about stresses to other parts of the body (such as upper arm, shoulders, neck or back).
7. You do several things to produce one (*product name*) and some of these things may be harder on your arm than others.
   I will ask you to rate:
   a. On the average or overall, how hard this job is on your arm.
   b. Give me a rating for the activity you find to be the hardest on your arm.

Position / Worker Specific Data Form Instructions

1. **Prior Work Experience Starting From Current Job**
   - **a.** I will ask you the overall or average level of stress you feel on your arm while performing your present job as well as performing previous jobs. (Remember, “arm” is from your elbow to fingertips).
   - **b.** Concentrate on your right/left (dominant) arm.
   - **c.** Please rate the overall or average level of physical stress you feel on your right/left arm for your current job including all job stations you rotate to.
   - **d.** What job were you performing before this job?
   - **e.** Please rate the overall or average level of physical stress on your right/left arm for your previous job(s).
   - **f.** Follow steps d and e for other previous jobs up to a total of a maximum of **5 previous jobs or 10 years of employment**.
2. **Standardized Grip Force (10Kg), Dominant Hand**
   - **a.** Please grip this device and **slowly** increase your force until the pointer covers the white area (Shoulder 0º, Elbow 90º). Keep holding it until I ask you to relax.
   - **b.** Concentrate on your right/left (dominant) arm. (Remember, arm is hand/wrist/forearm/elbow). I will ask you to rate the level of physical stress you feel in your dominant arm.
   - **c.** Let the worker apply force for 3-4 seconds.
   - **d.** Please rate the level of physical stress on your arm.
3. **Beginning and End of Shift Ratings**
   - a. Some people feel the same stress on their hand/wrist/forearm/elbow throughout the shift. Others feel different levels of physical stress at the beginning and end of a shift (job rotation included).
   - b. Concentrate on your dominant arm.
   - c. Please rate the overall or average level of physical stress on your arm at the beginning of your work shift on a typical workday. (Remember, “arm” is from your elbow to your fingertips.)
   - d. Please rate the overall or average level of physical stress on your arm at the end of your work shift on a typical workday. (Remember, “arm” is from your elbow to your fingertips.)

What is an Exertion
Force Level Ratings

1. Movements at the elbow or shoulder do not count as an exertion.

2. Exertion is gripping, pinching, holding weights applying force for example pushing,/pulling something with the hand (button pushing)

3. Exertion should affect the muscles on the forearm or the intrinsic muscles in your hand.

4. In a continuous cyclic manner, wrist flexion and then extension counts as ONE exertion.

5. Forearm rotation for example pronation and supination is ONE exertion. This is treated different than forearm rotation.

6. Picking up something with palm down using a palmer grasp in a continuous motion, quickly turn palm up (grasp does not change is ONE exertion not 2 exertions. However, if you grasp something with palm down and hold it for 1 second or more and turn your palm up, then there are two exertions (note the 2 different force levels).

7. Inspection Task- You twist your wrist (flexion, extension, ulnar deviation, radial deviation) this is ONE exertion. Again you twist your wrist = 2nd exertion. If the pause in motion is more than 1 second and flexion to extension is greater than 45° this is a separate exertion.

8. Pushing and pulling motion normally occurs at the elbow or shoulder. You are only grasping the object or maintaining contact with the object. All this motion is ONE exertion which is grasping.

9. Lifting between floor and waist height is low stress on the wrist. Therefore, the Borg rating should reflect this point.

10. **Regrasp**- Start with pinching something, for example taking something out of a tray, then if you quickly flip/toss the object up into the hand and change it to a power grasp, this would be considered TWO exertions.
    1. Pinching
    2. Other power grasp

11. For the purpose of exertion there are 2 types of grips, one is pinch (2 point, 3 point 4 point, palmer, lateral pinch, scissor pinch) and the other is grasp (power, hook, palmer, oblique). Do not differentiate between the different types of pinch or different types of grasp.

    a. When holding a part/tool, in one hand (i.e. open hand straightening of wires) that item is less than 1lb. Borg rating will = 0 and exertion and duration will not be counted. If item is greater than 1 lb. the Borg rating is 0.5.
    b. If a part/tool is being held with a controlled grasp and regardless of whether the tool is being used, exertion is one with a Borg rating of 1.

Force Level Ratings

1. Use field 9 on Job Specific Data Form if available to help identify major sub tasks.

2. Peak force sub task should not be lower then the lowest value of field 15h or 15i. In general the peak subtask should equal the force rating of field 15h.

3. When you fill out the form you need to fill out the task description in detail!
